# Association of *FCRL3* gene variants with rheumatoid arthritis susceptibility in the indian population: a combined case-control and *in- silico* analysis

**DOI:** 10.3389/fbinf.2026.1809854

**Published:** 2026-06-03

**Authors:** Mohamed Muzammil S, Asha Devi S

**Affiliations:** Department of Biomedical Sciences School of Bio Sciences and Technology, Vellore Institute of Technology, Vellore, Tamil Nadu, India

**Keywords:** FCRL3, rheumatoid arthritis, rs7528684, rs7522061, rs11264799, rs264797, rs7549100, SHP-1

## Abstract

**Background:**

Rheumatoid Arthritis (RA) is characterized as a systemic, chronic inflammatory autoimmune condition that causes joints damage and bone deformities. Recent studies indicate that the Single Nucleotide Polymorphisms (SNPs) within the immunoregulatory genes like Fc receptor-like3 (*FCRL3*) have a significant association with RA. The primary objective of this study is to explore the association between *FCRL3* gene variants and RA risk in 226 Indian RA patients compared to 239 controls.

**Methods:**

Genotyping of *FCRL3* SNPs rs7522061, rs11264799, rs11264797, and rs7549100 was performed using High-Resolution Melting Analysis, followed by Sanger sequencing to validate the accuracy of the results. To assess the association between SNPs and RA, unadjusted odd ratios (ORs) with 95% Confidence Interval (CI), gender-stratified analyses, and multivariable logistic regression adjusted for age and sex were performed. Furthermore, to investigate the impact of rs7522061 SNP in RA pathogenesis, an *in silico* approach involving homology modelling and molecular docking (HADDOCK) of FCRL3 wild type vs. variant (N28D) protein with SHP-1 was employed and 1,000 ns?Coarse-Grained Molecular Dynamics (CG-MD) simulations (GROMACS/MARTINI v3.0) was performed.

**Results:**

The rs7522061 G/A genotype was significantly associated with increased RA risk (unadjusted OR = 1.84, 95% CI = 1.21–2.79, adjusted *p =* 0.0200). Multivariable logistic regression confirmed this association was independent of age and sex (adjusted OR = 2.03, *p =* 0.0028). *In-silico* analysis revealed that the FCRL3 N28D variant formed a more favourable docking interface with SHP-1 (mean HADDOCK score: 61.1 ± 11.0 vs. - 53.45 ± 5.84) compared to wild type and 1,000 ns CG-MD stimulation exhibited faster equilibration, lower RMSD, tighter Interfacial distance (0.15 nm), more persistent interprotein contacts, and greater compactness.

**Conclusion:**

*FCRL3* SNP rs7522061 G/A genotype contributes to RA susceptibility in the Indian population. The computational study suggests that the N28D substitution enhances FCRL3–SHP-1 complex stability, which may disrupt Treg function through increased negative signalling.

## 1 Introduction

Rheumatoid Arthritis (RA) is characterized as a persistent chronic autoimmune condition distinguished by prolonged synovitis that leads to cartilage damage, bone erosion, and debilitating joint deformity (Lin et al., 2020; Said et al., 2021). Globally, the estimated prevalence of RA ranges between 0.4% and 2%. The incidence of RA follows a gender-based pattern, characterized by a female to male ratio of 3:1 (Muravyev, 2018; Lin et al., 2020). The etiological landscape of RA is highly intricate due to the interplay between genetic susceptibility and non-genetic factors such as environmental exposures, lifestyle choices, and patient demographics (Ye et al., 2021; Goronzy et al., 2010; Liao et al., 2009). Exploring the genetics of RA is critically important, as genetic variations are responsible for 65% of disease’s heritability (Akhtar et al., 2021). Single Nucleotide Polymorphisms (SNPs), are single-base variants present in more than 1% of the population, represent the most frequent form of genomic diversity, which are the primary drivers of inter-individual genetic variability. About 50% of SNPs occur within the coding regions of gene which includes 25% of synonymous and 25% of missense variants (Akhtar et al., 2020; Akhtar et al., 2021). SNPs occurring in the non-coding region may alter the mRNA stability or influence the gene’s promoter activity, which leads to altered gene expression (Shastry, 2009). Thus, experimental investigations are important to explore the SNPs and their key role in RA risk, specifically how they influence biological pathways and the mechanisms leading to the autoimmunity.

Fc receptor-like 3 (*FCRL3*), an immunoregulatory gene, expressed predominantly as a transmembrane protein on regulatory T cells (Tregs), and at moderate levels on other immune cells. FCRL3 is a member of the immunoglobulin receptors, which serves as an immune regulator by modulating Treg immunosuppressive activity. Therefore, genetic variations within the gene are consistently linked with an increased predisposition to autoimmune diseases. Specifically, SNPs within the *FCRL3* gene are significantly linked with RA susceptibility across global populations (Kochi et al., 2005; Kalantar et al., 2020; Zhong et al., 2019; Gu et al., 2015). The *FCRL3* gene located on chromosome 1q21 spans 24.3 kb of DNA, which comprises of 16 exons and 15 introns. Its protein consisted of 734 amino acids: residues 1–17 act as a signal peptide, residues 18-573 form six immunoglobulin-like domains in the extracellular region, residues 574–594 links extracellular and cytoplasmic tail by a transmembrane region and residues 595-734 form the cytoplasmic tail. The cytoplasmic tail contains two distinct signalling motifs, namely, Immunoreceptor Tyrosine-based Inhibition Motif (ITIM) and Immunoreceptor Tyrosine-based Activation Motif (ITAM), which are encoded by exon 15 and 13 respectively (Plewa et al., 2025; Li et al., 2013; Chistiakov and Chistiakov, 2007). In most tyrosine-based signalling pathways, these motifs enable the protein to serve as both an activator and an inhibitor (Zhang et al., 2015; Bahabayi et al., 2024). Studies suggest that ITAM and ITIM ensure regulated immune responses by preventing an inappropriate activation and inhibition of Tregs, a mechanism vital for maintaining self-tolerance and preventing autoimmunity (Agarwal et al., 2020; Bajpai et al., 2012) (Xu et al., 2002). Tregs are specialized subsets derived from CD4^+^ T lymphocytes that are important for maintaining peripheral immune homeostasis and preventing autoimmune responses. Tregs exert their immunosuppressive role through multiple mechanisms: notably cytokine-mediated suppression, contact-dependent cytolysis of the target cells, metabolic disruption of the local environment, and modulation of Antigen-presenting cells (APC) (Goswami et al., 2022; Vignali et al., 2008). For Treg suppressive function, the TCR (T Cell Receptor) of the Treg must engage with a peptide displayed by MHC (Major Histocompatibility Complex) class II molecule on APC. The TCR exists as an αβ heterodimer which is non-covalently linked with CD3 complex (γ, δ, ε chains, and ζζ homodimers) since TCR lacks intrinsic signalling capability. The TCR-CD3 complex serves as an essential molecular interface for recognizing peptide-MHC ligand complex and processing the suppressive signals in Tregs. Upon activation, this complex initiate tyrosine-based phosphorylation events mediated by SRC (LCK and FYN), and SYK (ZAP70) family kinases. These kinases subsequently phosphorylate ITAM within the cytoplasmic domain of TCR-associated CD3 protein ζ-chains (Chistiakov and Chistiakov, 2007). This is the initiating step of TCR signalling, and this signalling cascade enables the immunosuppressive function of Tregs by synthesis and release of immunosuppressive cytokines, which in turn reduces the auto-reactive immune cells population from periphery, thereby preventing autoimmunity (Vahl et al., 2014; Moran et al., 2011). Therefore, any compromise or dysfunction in Treg activity results in a loss of peripheral tolerance, which leads to uncontrolled activation of auto-reactive immune cells, which directly contribute to the development of autoimmune disorders, including RA (Goswami et al., 2022; Schmidt et al., 2015; Rostamzadeh et al., 2018). In recent studies, a consistent correlation was observed between elevated FCRL3 protein levels (due to SNP) on the surface of Tregs and subsequent impairment in suppressive function (Bajpai et al., 2012; Agarwal et al., 2020). The underlying molecular mechanism governing FCRL3-mediated inhibition of Treg immunosuppressive function is highly dependent on its ITIM. Upon receptor ligation, such as by binding to secretory IgA, the tyrosine residues (Tyr692 and Tyr722) within ITIM undergoes phosphorylation (Xu et al., 2002; Chistiakov and Chistiakov, 2007; Agarwal et al., 2020). The phosphorylated ITIM create specific docking interfaces that exhibit high binding affinity for the SH2-domain containing protein tyrosine phosphatases (PTPs), most notably SHP-1 (PTPN6) and to a lesser extent for SHP-2 (PTPN11).

SHP-1 is a cytoplasmic protein tyrosine phosphatase, also known as PTPN6 or SH-PTP1. Structurally the phosphatase contains 595 amino acids which are organized into three primary functional regions: an N-terminal segment containing two tandem SH2 domains (N-SH2: residues 4–103; C-SH2: residues 112–210), a central catalytic PTP domain (residues 216–526), and a short C-terminal tail (residues 527–595). The tandem SH2 domains facilitate high-affinity and site specific binding to phosphorylated tyrosine residues within ITIM of FCRL3. Upon receptor ligation to FCRL3, the C-SH2 domain exhibits stronger binding affinity to the phosphorylated Tyr692 ITIM motif compared to the N-SH2 domain. This interaction relieves autoinhibition of SHP-1, which fully activates its phosphatase domain to dephosphorylate key TCR signalling molecules, including LCK (Tyr-394) and ZAP-70 (Tyr-493), thereby attenuating downstream signalling and modulating Treg suppressive function (Kochi et al., 2009, Lorenz, 2008, Wang et al., 2012, Craggs and Kellie, 2001). Although multiple studies have confirmed that both phosphatases (SHP-1 and SHP-2) interact with FCRL3 ITIM, SHP-1 delivers potent inhibitory signalling within Tregs, and its dysregulation is more consistently linked with RA pathogenesis compared to other phosphatase (Bottini and Stanford, 2015; NCADV, 2017; Xu et al., 2002; Maine et al., 2012; Jassim et al., 2022).

Multiple studies have established that *FCRL3* SNPs are linked with the risk of developing RA across diverse populations (Owen et al., 2007; Yang et al., 2021; Bajpai et al., 2012). Despite established associations, we still lack empirical data on the functional validation of these SNPs, especially, the non-synonymous/missense SNP rs7522061 (A>G) located within the 4^th^ exon region of *FCRL3* gene, which leads to an amino acid substitution from asparagine to aspartic acid (N28D) at 28th amino acid position in the FCRL3 protein. The functional impact of this variant on FCRL3 protein remains unexplored (Matesanz et al., 2008; Owen et al., 2007). Another synonymous SNP rs11264799 found within the *FCRL3* regulatory region has been link to autoimmune diseases and two intronic variants rs11264797 and rs7549100 remain unexplored for their association with RA (Zhong et al., 2019; Zhang et al., 2020). The RA patients within the Indian cohort were not screened for all these four SNPs. Therefore, this study intents to identify the frequency of these four SNPs and their association with RA susceptibility in the Indian population. Furthermore, we hypothesise that the non-synonymous SNP rs7522061 (A>G) may structurally modify the FCRL3 protein. This missense variant substitutes asparagine (N) with aspartic acid (D) at position 28 (N28D) in the extracellular Ig-like domain. Substituting the neutral, polar asparagine with a smaller, negatively charged aspartic acid is predicted to modify the local electrostatic potential and backbone conformation. This change could propagate allosterically through the transmembrane region, potentially reorienting the intracellular ITIM or modulating their accessibility, thereby creating a more favourable docking interface for the SHP-1 protein. This may facilitate enhanced FCRL3-SHP-1 complex formation within Tregs. This strengthened interaction could amplify the negative regulatory effect of SHP-1 on the TCR signalling pathway, potentially leading to impaired Treg function and loss of peripheral tolerance, thereby predisposing to the development of RA and other autoimmune conditions. To prove this hypothesis an *in silico* study was carried out which scrutinized the binding affinity between the wild-type and variant (N28D) FCRL3 protein with SHP-1. These protein-protein interactions were accomplished through molecular docking and Molecular Dynamic (MD) simulations to provide a comprehensive understanding of their inhibitory signalling.

## 2 Methodology

### 2.1 Sample collection and genomic DNA isolation

The patients with RA from various regions across India who came to receive treatment at Sri Narayani Hospital and Research Centre, Vellore, Tamil Nadu, India, participated in this study. About 5 mL of peripheral blood samples were drawn from 239 healthy controls and 229 R A patients of both sexes, with an age range of 30–60 years. The sample collection procedures were carried out under the oversight and approval of the ethical clearance (IEC/IRB No.29/08/07/2022) from the Institution with informed consent. Inclusion/exclusion criteria were followed according to the 2010 ACR/EULAR criteria, and the demographic and clinical characteristics of the study participants were mentioned in Table 1. From collected blood sample, total genomic DNA was purified *via* Miller’s salting-out technique, a simple, cost-effective technique for high-quality DNA purification from biological samples (Miller et al., 1988). Nanodrop spectrophotometer was employed to evaluate the yield and purity of the DNA samples (Desjardins and Conklin, 2010). Post-hoc power estimations were determined using G^*^ Power software (v3.1.9.7) to validate the cohort’s statistical power. For a total sample size of 465 (239 healthy volunteers and 226 R A cases), the study achieved a statistical power of 0.99 (99%) at α = 0.05 for a two-tailed chi-square test, indicating adequate sample size to detect associations with low risk of Type II error.

**TABLE 1:** Demographic and clinical characteristics of study participants.

Characteristics	RA (n = 226)	Controls (n = 239)
Sex: Female/Male	144/82	132/107
Age (mean ± SD)	48 ± 11	47 ± 12
Ethnicity	Indian	Indian
Duration of disease (years, mean ± SD)	3 ± 1	NA
ESR positivity, *n* (%)	149 (65.9%)	NA
Anti-CCP positivity, *n* (%)	167 (73.8%)	NA
CRP positivity, *n* (%)	189 (83.6%)	NA
Drugs: NSAID/DMARD	0/0	NA
DAS28 score (mean ± SD)	3 ± 1	0 ± 0

### 2.2 Genotyping quality control and STREGA compliance

Genotyping quality control and reporting were performed in accordance with the STREGA (STrengthening the REporting of Genetic Association studies) guidelines. A detailed participant flow according to STREGA recommendations is provided in Supplementary Tables 2, 3. To address potential population stratification, the following measures were implemented: (1) both RA cases and healthy controls were recruited from the same hospital (Sri Narayani Hospital and Research Centre, Vellore), which receives patients from various regions across India; (2) only individuals with self-reported Indian ethnic background were included, with preference given to participants sharing similar ancestral origins where possible; (3) age and sex were adjusted as covariates in the multivariable logistic regression model for SNP that showed significant association in the initial analysis; and (4) careful matching was attempted during sample collection.

### 2.3 *FCRL3* gene amplification and SNP genotyping

The isolated DNA samples were brought to uniform concentration (100 ng/μL) and used as a template to amplify SNP (rs7522061, rs11264799, rs11264797, and rs7549100) target regions within the *FCRL3* gene. Specific forward and reverse primers were designed based on the reference sequence retrieved from the NCBI database to flank the target region of each SNP with different amplicon lengths such as 441 bp (rs7522061), 465 bp (rs11264799), 502 bp (rs11264797) and 480 bp (rs7549100) (Accession Number (AN): NG_023241). Each Polymerase Chain Reaction (PCR) mixture used contains 4 µL of DNA sample as template, 2 µL of each primer (forward and reverse at working concentration of 10 pmol/μL), 4 µL of 5X buffer, 0.2 µL of PrimeSTAR HS DNA polymerase (with proof reading activity) and 7.8 µL of nuclease-free water to reach a total volume of 20 µL. The amplified products were visualized using a gel documentation system. High-Resolution Melting Analysis (HRMA), a high-throughput molecular method, was utilized for genotyping the variants (Vossen et al., 2009). For genotyping, PCR amplicons were diluted at 2:8 ratio and used as a template. HRMA reaction mixture for each SNP was prepared by combining 2 µL of DNA template, 1 µL of each primer (forward and reverse at working concentration of 5 pmol/μL), 5 µL of Qiagen Eva green master mix, finally adjusted the volume to 10 µL by adding nuclease-free water. The internal primers utilized for genotyping SNPs are mentioned in Table 2. The PCR and qPCR/HRMA reaction conditions are detailed in Supplementary Table S1. Duplicate analysis was conducted for each sample and the HRMA melt curves were processed through the Bio-Rad Precision Melt Analysis software. Samples exhibiting variations *via* melt curves were cross validated by Sanger’s sequencing to authenticate SNPs at the respective loci (Shri Preethi and Asha Devi, 2022).

**TABLE 2:** Primers used to amplify *FCRL3* gene and genotyping SNPs.

SNP ID	Variant	Region	Initial PCR primers	HRMA genotyping primers
rs7522061	A > G	Exon 4	F: 5’ – CTGACTGTAGGTTGGGCTC - 3′ R: 3’ - TGCATCAATCCCCATGTCTGC - 5′	F: 5′- CCCCAAAAGCTGTACTTCTC - 3′ R: 3′- GCTAGGGAATGTGATATGCTG - 5′
rs11264799	G > a	5′UTR	F: 5’ – GACACGAAAGCAAACAAGG - 3′ R: 3’ – CCCCTTCACTACCTTGTC - 5′	F: 5′- CCTGTTCAGAAGCCTCATAAC - 3′ R: 3′- GCGTGGTGTGAATCACACAG - 5′
rs11264797	G > T	Intron	F: 5′- GCCTATTTGTACCCCTA - 3′ R: 3′- ATTGAGCAAACATGGACA - 5′	F: 5′- GTCTGCCTTTAAAATGAGG - 3′ R: 3′- CACATTTACCCCTTACATC - 5′
rs7549100	T > C	Intron	F: 5′- CCTGGTTGAATCACTTAC - 3′ R: 3′- TGACAGGATCTGTACCCC - 5′	F: 5′- GATATGATTAGGTTTCATGTC - 3′ R: 3′- GTTATGGCTATTATACTAAC - 5′

F, forward; R, reverse; UTR, untranslated region.

### 2.4 FCRL3 protein’s structural modelling and insertion of variant

A 3D model of the candidate protein was computationally simulated as no crystallized structure of FCRL3 presently available in Protein Data Bank (PDB) (https://www.rcsb.org/). A three-dimensional model of the full-length human FCRL3 protein was generated through template-based homology modelling using SWISS-MODEL server. When the FCRL3 sequence was submitted to the server, no suitable high-identity experimental template was identified. The closest homologous template (Contactin-2, PDB 8k53.1.A) showed only 20.4% sequence identity, which is too low for reliable homology modelling. Therefore, we selected the AlphaFold Database model of FCRL3 (Q96P31.1.A) provided by SWISS-MODEL as the best available structure. This model showed comparatively good quality metrics with a GMQE score of 0.77% and 100% sequence coverage (Biasini et al., 2014). Stereochemical quality and structural reliability of generated FCRL3 protein model was analysed *via* Ramachandran plot analysis, and the final 3D model of FCRL3 protein was visualized using PyMOL software. The amino acid substitution at the 28th position (N28D), resulting from SNP rs7522061 was introduced into the FCRL3 protein using PyMOL software to generate the variant/N28D FCRL3 protein model (Biasini et al., 2014; Rosignoli and Paiardini, 2022).

### 2.5 *In-silico* assessment of SNP rs7522061 N28D effect on FCRL3 protein

To investigate the structural stability, dynamics and potential allosteric signalling of FCRL3 N28D variant, the DynaMut2 server was utilized. This server integrates Normal Mode Analysis (NMA) with graph-based signatures to quantify the impact of variant on the protein’s global dynamics and overall stability across the entire scaffold. By characterising alterations in conformational flexibility, DynaMut2 facilitates the identification of long-range allosteric effects that propagate through protein structure. The change in folding free energy change (ΔΔG) between the wild type and N28D were used as a primary metric for thermodynamic stability. Substitutions yielding ΔΔG >0 were classified as stabilizing, while those with ΔΔG <0 were categorised as destabilizing (Joshi et al., 2025). This approach enabled a comprehensive mapping of the N28D variant’s influence on the FCRL3 protein energy landscape. Furthermore, the deleterious effect of N28D on FCRL3 protein was predicted using the following online computational tools: 1. Combined Annotation Dependent Depletion (CADD); Which uses a machine learning approach that integrates with more than 60 genomic features to analyse genetic variations. CADD assigns a C-score to each variant, where a high score indicates a greater likelihood of a variant being deleterious (Rentzsch et al., 2019), 2. PolyPhen-2; This tool predicts the functional impact of non-synonymous SNPs on structure integrity of protein. It assigns a score ranging from 0.0 (benign) to 1.0 (deleterious). PolyPhen-2 determines the functional annotations of the non-synonymous SNPs, which mapping the coding variants to a specific gene transcript to evaluate their impact on protein amino acid sequence and structural integrity. This information is used to generate conservation profiles that predict deleterious effect of missense SNPs (Adzhubei et al., 2013), 3. Sorting Intolerant From Tolerant (SIFT); SIFT estimates the potential impact of an amino acid substitution caused by SNPs (Sim et al., 2012).

### 2.6 Molecular docking and simulation

The structural interactions between the proteins were executed using HADDOCK (version 2.4) web server, which is specifically designed to incorporate experimental and bioinformatic data as constraints. This flexible docking approach was applied to map the binding interfaces of wild-type and FCRL3 N28D proteins with SHP-1 (Van Zundert et al., 2016). The SHP-1 protein’s 3D model was retrieved from the RCSB PDB (AN: 3PS5). The active residues for docking were identified using pocket prediction tools (P2Rank version 2.5 and CASTp version 3.0). The active binding site residues within the FCRL3 protein were defined as Val690, Leu691, Tyr692, Leu695, Asn721, and Tyr722. For SHP-1, the active residues included: Tyr276, Ile279, Glu355, Lys360, Asp419, Gly421, Ser453, Ser454, Ala455, Gly456, Ile457, Gly458, Arg459, Gln500, Thr501, and Gln504. Passive residues for both proteins were automatically selected by HADDOCK server (Kochi et al., 2009; Xu et al., 2002).

Docking results including the HADDOCK score, Van der Waals energy, Electrostatic energy, and Desolvation energy were obtained from HADDOCK server. Further to characterize the molecular interface, PDBsum server was used to visualize specific contacts, such as hydrogen bonds, disulfide bonds and salt bridges (Van Zundert et al., 2016). To investigate the stability and dynamic behaviour and to understand the functional consequences of the wild type and variant FCRL3 protein while interacting with SHP-1 protein, the docked complexes were subjected to Coarse-Grained Molecular Dynamic (CG-MD) simulation for 1,000 nanoseconds (ns) using GROMACS (version 2023.2) with the MARTINI force field. CG-MD simulation allows significantly longer simulation timescales (microsecond range) compared to all-atom MD, while maintaining reasonable accuracy for large protein-protein complexes. This approach was chosen to study the dynamic stability of protein-protein interactions, allosteric effects, and global conformational changes over extended periods. The atomistic structures of FCRL3 (wild-type and N28D) and SHP-1 were converted into coarse-grained representations using the standard MARTINI 3 mapping scheme, in which approximately four heavy atoms are represented by a single interaction bead based on their chemical properties (polarity, charge, and hydrophobicity). To maintain the structural integrity of the proteins during simulation, an elastic network model was applied to the backbone beads, introducing harmonic restraints between beads within a cutoff distance. The assembled protein-protein complex was placed in the center of a cubic simulation box with periodic boundary conditions. A minimum distance of 1.2 nm was maintained between the complex and the box edges. The system was solvated with MARTINI-compatible coarse-grained water beads and neutralized by adding counter-ions. Sodium (Na^+^) and chloride (Cl^−^) ions were added to achieve a physiological salt concentration of 0.15 M.

Energy minimization was performed to remove steric clashes and unfavourable interactions. The system was then equilibrated in two phases: first under the NVT ensemble (constant number of particles, volume, and temperature) at 310 K, followed by the NPT ensemble (constant pressure and temperature) at 1 bar. Following equilibration, the production CG-MD simulation was run for 1,000 ns? Non-bonded interactions were treated using the standard MARTINI cut-off schemes. Temperature and pressure were maintained at 310 K and 1 bar, respectively, throughout the production run to mimic physiological conditions. For the post-simulation analysis, various GROMACS tools were employed to process the trajectory data, especially, Root Mean Square Deviation (RMSD), Root Mean Square Fluctuation (RMSF), Solvent Accessible Surface Area (SASA), Radius of Gyration (Rg), number of interprotein contacts, minimum distance between proteins, centre-of-mass distance, and RMS deviation and cluster index analysis were calculated. The results were graphically plotted using XMGRACE software (Gopikrishnan and Doss, 2023; Kumari and Dalal, 2022; Premkumar et al., 2024; Kushwaha et al., 2021).

### 2.7 Statistical analysis

The allelic and genotype frequencies of the SNPs (rs7522061, rs11264799, rs11264797, and rs7549100) were compared between the RA cases and healthy controls using the unadjusted Chi square test (χ^2^).

To evaluate the association with RA; the unadjusted Odd ratio (OR) with 95% Confidence Interval (CI) was calculated from the 2 × 2 contingency tables using formula:

$$OR=\frac{a\times d}{b\times c}$$

Using Woolf’s method, the standard error of the log OR was calculated as:

$$SE\left\{\ln\left(OR\right)\right\}=\sqrt{\frac{1}{a}+\frac{1}{b}+\frac{1}{c}+\frac{1}{d}}$$

The *p-*value obtained from the Wald test (z = In (OR)/SE {in (OR)}).

Subsequently, the gender-stratified association analysis to evaluate risk associations independently for both male and female subgroups was investigated. To minimize statistical errors, multiple correction methods including Benjamini-Hochberg False Discovery Rate (BH-FDR) correction and Bonferroni correction were applied. A *p* value of *<0.05* was considered as the criterion for statistical significance (Shri Preethi and Asha Devi, 2022; Haynes, 2013).

### 2.8 Multivariable binary logistic regression analysis

Additionally, multivariable binary logistic regression was performed for the SNP rs7522061 which exhibited statistically significant after BH- FDR correction in the initial OR analysis. This analysis was conducted in R (version 4.5.1) and data were imported using the readxl package, and logistic regression models were constructed using glm function from the stats package. The model evaluated the association of SNP genotypes with RA risk while adjusting for potential confounders. Age and sex were included as covariates in the model to maintain the demographic imbalances between cases and controls. Analyses were conducted under four genetic models: (1) genotypic model (A/A as reference), (2) additive model (coded as 0/1/2 copies of G allele), (3) dominant model (G/A+ G/G vs. A/A), and (4) recessive (G/G vs. G/A+ A/A). Genotype data was converted into categorical data to assess non-linear relationship. ORs with 95% CIs were calculated using Wald tests, and *p*-values <0.05 were considered significant. Results are reported as regression coefficients (B), standard error (SE), and Wald statistics. The complete analysis script is provided in the Supplementary Material 1 to ensure full reproducibility.

## 3 Results

**FIGURE 1:**
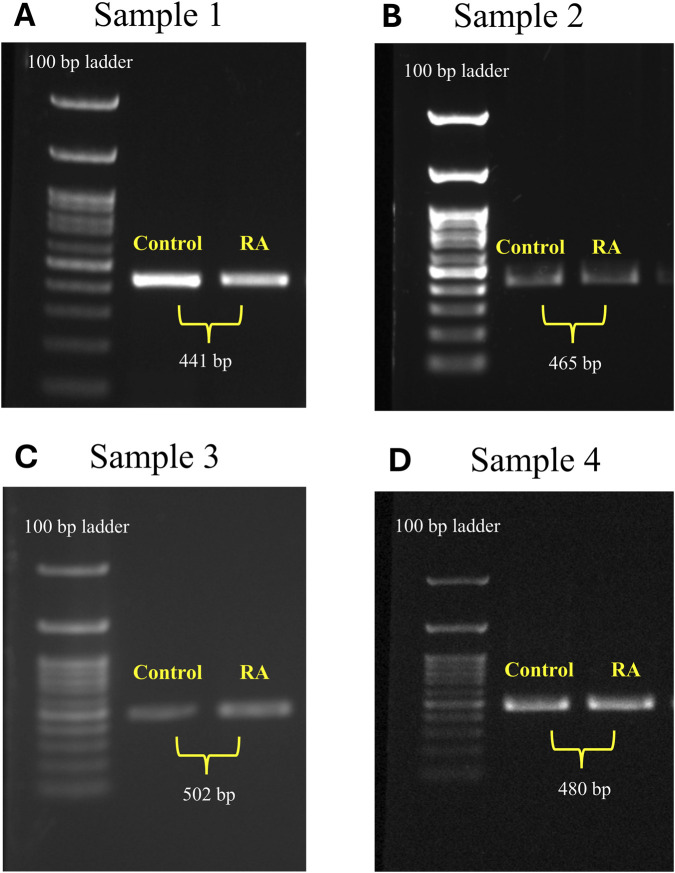
PCR amplicons of *FCRL3* SNPs target regions: **(A)** rs7522061 (441 bp) (sample1). **(B)** rs11264799 (465 bp) (sample 2). **(C)** rs11264797 (502 bp) (sample 3). **(D)** rs7549100 (480 bp) (sample 4) obtained from controls and RA patients.

**FIGURE 2:**
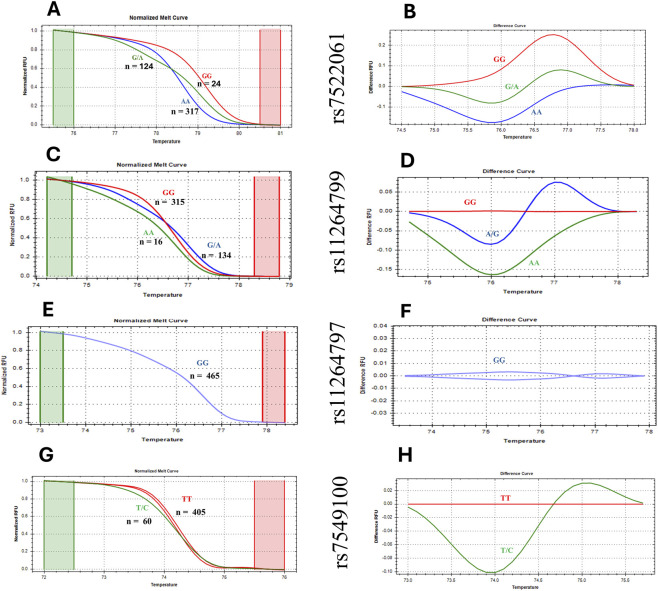
Normalized and difference melt curves for each SNP with their genotypes count (n = controls and RA): **(A, B)** for SNP rs7522061 genotypes (G/G, G/A, and A/A), **(C, D)** for SNP rs11264799 genotypes (G/G, A/G, and A/A), **(E, F)** for rs11264797 genotypes (G/G), and **(G, H)** for rs7549100 genotypes (T/T and T/C).

**FIGURE 3:**
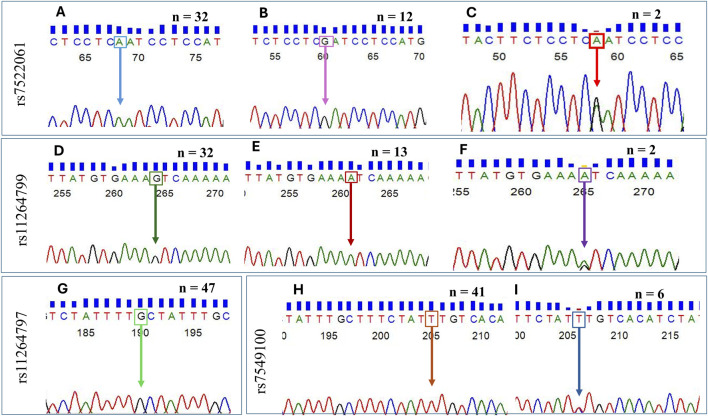
Representative Sanger sequencing chromatograms for each observed FCRL3 SNP genotypes with their corresponding DNA sequences. SNP rs7522061: **(A)** Wild type - A/A, **(B)** Homozygous variant - G/G, **(C)** Heterozygous - variant G/A; SNP rs11264799: **(D)** Wild type - G/G, **(E)** Homozygous variant - A/A, **(F)** Heterozygous variant - A/G; SNP rs11264797: **(G)** Wild type - G/G; SNP rs7549100: **(H)** Wild type - T/T, **(I)** Heterozygous variant - T/C.

To screen *FCRL3* SNPs, rs7522061, rs11264799, rs11264797, and rs7549100 in the Indian population, the genomic DNA was extracted from all blood specimens obtained from study subjects (239 healthy controls and 226 R A patients). The SNP target regions within *FCRL3* gene was amplified using PCR. Figures 1A–D displays the amplified regions of four specific *FCRL3* gene regions (SNPs rs7522061, rs11264799, rs11264797, and rs7549100) with the fragment sizes of 441, 465, 502, and 480 bp, respectively. The resultant amplicons served as a template for genotyping the SNPs using HRMA technique. HRMA exhibited three distinct genotype clusters delineated by normalized and difference melting curves. Figures 2A–H illustrate the normalised and differential curves of each SNPs, and each melt curve represents the presence of different genotypes. Further, to identify the genotype associated with melt curve, the samples showing different melt curves were validated through Sanger sequencing. The sequencing results of SNP rs7522061 (Figures 3A–C) indicated the existence of A/A (wild type), G/G (homozygous variant), and G/A (heterozygous variant) in the rs7522061 base pair window. Figures 3D–F represent the presence of G/G (wild type), A/A (homozygous variant), and A/G genotype (heterozygous variant) in the rs11264799 loci. Figure 3G depicts the presence of only wild type (G/G) genotype in the rs11264797 loci. Figures 3H,I indicate the existence of T/T (wild type) and T/C (heterozygous variant) in the rs7549100 loci on *FCRL3* gene.

### 3.1 Statistical analysis and quality control

#### 3.1.1 Unadjusted association analysis

Initial, screening for *FCRL3* SNPs was performed using unadjusted Chi-square (χ^2^) and association metrics, including OR with 95% CI, were derived from 2 × 2 contingency tables using the Woolf’s method for standard error and Wald’s test for *p-value* estimation. Among the variants tested, only rs7522061 demonstrated a significant association with RA susceptibility in the crude analysis (Table 3). Using the wild-type A/A genotype as reference, the heterozygous G/A genotype conferred a significantly increased risk of RA (OR = 1.84, 95% CI = 1.21–2.79, adjusted *p =* 0.0200, and χ^2^ = 8.28), while the homozygous G/G genotype showed no significant association (OR = 1.247, 95% CI = 0.51 to 2.99, adjusted *p =* 1.000 and χ^2^ = 0.245). No significant association was observed for rs11264799 genotypes, including heterozygous G/A (OR = 1.515, 95% CI = 1.01 to 2.26, adjusted *p* = 0.2165 and χ^2^ = 4.082) and homozygous genotype A/A (OR = 1.374, 95% CI = 0.50 to 3.55, adjusted *p* = 1.000 and χ^2^ = 3.871). For rs7549100, the minor C allele was observed only in the heterozygous T/C form (no C/C homozygotes were detected), this genotype showed no significant association with RA risk (OR = 1.4507, CI = 0.83 to 2.50, adjusted *p* = 0.905, and χ^2^ = 1.7897). The SNP rs11264797 was found to be monomorphic in the Indian cohort (100% homozygosity G/G, Minor Allele Frequency (MAF) = 0.00).

**TABLE 3:** Association analysis between *FCRL3* SNPs genotype and RA risk.

SNP ID	Genotypes	*Controls n (%)*	*RA n (%)*	*χ* ^*2*^	OR	95% C. I	Z-stats	*Adj p-value*	*FDR%*	*HWE p-value (controls)*
rs7522061	A/A (ref)	181 (75.7)	136 (60.2)	-	1.0	-	-	-	-	0.0613
	G//A	50 (20.9)	74 (32.7)	8.2850	1.840	1.21 to 2.79	2.86	0.0200	0.0200	
	GG	8 (3.4)	16 (7.1)	0.2450	1.247	0.51 to 2.99	0.495	1.000	0.6206	
rs11264799	G/G (ref)	173 (72.4)	142 (62.8)	-	1.0	-	-	-	-	0.4760
	G/A	59 (24.7)	75 (33.2)	4.0822	1.515	1.01 to 2.26	2.017	0.2165	0.1082	
	A/A	7 (2.9)	9 (4)	0.3871	1.375	0.50 to 3.55	0.621	1.000	0.6206	
rs7549100	T/T (ref)	213 (89.1)	192 (84.9)	-	1.0	-	-	-	-	0.3739
	T/C	26 (10.9)	34 (15.1)	1.7897	1.450	0.83 to 2.50	1.334	0.9050	0.3017	

#### 3.1.2 Covariate-adjusted logistic regression

To mitigate confounding by demographic variables, multivariable binary logistic regression was performed for the significant variant, rs7522061. Under the genotypic model (A/A as reference) and after adjustment for age and sex as covariates, the G/A genotype maintained a strong and statistically significant association with increased RA risk (adjusted OR = 2.031, 95% CI = 1.27–3.23, and *p =* 0.0028). The homozygous variant G/G genotype did not reach statistical significance in the adjusted model (adjusted OR = 2.48, 95% CI = 0.95–6.50, and *p =* 0.0634). Among the covariates, age was a significant predictor of RA risk (adjusted OR = 1.089, 95% CI = 1.06–1.11, p < 0.0001), whereas sex was not (adjusted OR = 0.720, 95% CI = 0.47–1.08, p = 0.1182) (Table 4).

**TABLE 4:** Multivariable binary logistic regression analysis of rs7522061 genotypes and RA risk (genotypic model with A/A as reference), adjusted for age and sex.

Variable	B (coeff)	SE	Wald (z2)	Adjausted OR	95% CI	*p-value*
A/A (reference)	-	-	-	1.00	-	-
G/A	0.7086	0.2371	8.929	2.031	1.27–3.23	0.0028
G/G	0.9110	0.4907	3.447	2.487	0.95–6.50	0.0634
Age	0.0852	0.010	64.85	1.089	1.06–1.11	0.0001
Sex	−0.3287	0.210	2.441	0.720	0.47–1.08	0.1182

#### 3.1.3 Multiple-testing correction

To account multiple independent statistical tests and to control the risk of false positives, two complementary correction methods were applied to the univariate genotype association analyses. First, the BH-FDR procedure was used, as it is the most appropriate method when testing several SNPs to maintain reasonable statistical power. The raw *p-*values for the genotype comparisons (rs7522061 G/A, rs7522061 G/G, rs11264799 G/A, rs11264799 A/A, and rs7549100 T/C) were adjusted using the BH method. After correction, only the heterozygous G/A genotype of rs7522061 remained statistically significant at FDR <0.05 (adjusted p = 0.0200). In addition, a more conservative Bonferroni correction was applied (family-wise error rate control), in which the significance threshold (α = 0.05) was divided by the number of independent tests performed (5 genotype comparisons). This yielded an adjusted significance threshold of *p <* 0.05. Only the rs7522061 G/A genotype survived this stringent correction as well. These dual approaches (BH-FDR and Bonferroni correction) ensure robust control of Type I error while providing both powerful and conservative interpretations of the genetic associations.

#### 3.1.4 Gender-stratified analysis

To evaluate whether gender modulates the association between *FCRL3* SNPs and RA risk, a stratified analyses were performed (Supplementary Table S6). In the female subgroup, the heterozygous G/A genotype of rs7522061 showed a nominal significance (OR = 1.732, 95% CI 1.02–2.92, *p =* 0.0435, and χ^2^ = 4.0745). A comparable size effect was observed in the male subgroup (OR = 1.895, 95% CI 0.94–3.81, *p =* 0.0642, and χ^2^ = 3.4260), though it did not reach the threshold for significance. Following correction through multiple testing, the associations in both stratified subgroups became non-significant. However, when the cohort was analysed as a whole (pooling both genders), the rs7522061 G/A genotype maintained significant associated with RA susceptibility (*p =* 0.020). No significant association was observed between genders for the other SNPs screened (rs11264799, rs11264797, and rs7549100). These findings demonstrate that the association between the rs7522061 G/A genotype and RA risk is independent of gender. The overall (pooled) analysis provides greater statistical power to detect the genetic effect, while the direction and magnitude of the association remain consistent across both genders.

#### 3.1.5 Hardy–weinberg equilibrium (HWE) and allelic distribution

HWE is a principle stating that allele and genotype frequencies remain stable across generations in the absence of evolutionary pressures. In clinical studies, testing HWE in the control group is a vital quality control step; significant deviations suggest potential genotyping errors, sample relatedness, or population stratification. To assess HWE, a Chi-square goodness-of-fit test is employed to compare the observed genotype counts against expected values. A *p -*value >0.05 indicates that the population is in equilibrium, validating the genetic integrity of the study cohort. All investigated SNPs in the control group were consistent with HWE (*p >* 0.05), validating the genetic stability of our study population and confirming the absence of significant genotyping errors or population stratification. For the significant variant rs7522061, the control distribution (A/A = 181, G/A = 50, G/G = 8) yielded HWE *p =* 0.0613, with a major allele (A) frequency of 0.86 and a MAF (G) of 0.14. Similarly, for rs11264799, the distribution (G/G = 173, G/A = 59, A/A = 7) was in equilibrium (*p =* 0.4760), with a major allele (G) frequency of 0.85 and a MAF (A) of 0.15. The intronic variant rs7549100 (T/T = 213, T/C = 26, C/C = 0) also adhered to HWE (*p =* 0.3739), showing a major allele (T) frequency of 0.95 and a MAF (C) of 0.05. Finally, rs11264797 exhibited 100% homozygosity (G/G) in the control group, resulting in allele frequency of 0.00 (Table 3).

#### 3.1.6 Sensitivity analyses

Sensitivity analysis (exclusion of G/G homozygotes) is a standard method to ensure the robustness of associations. We repeated the analysis after excluding G/G individuals, leaving only A/A and G/A genotypes (n = 441). The heterozygous G/A genotype remained significantly associated with increased RA risk in the full cohort (OR = 1.96, 95% CI = 1.29–3.00, *p =* 0.0015, and χ^2^ = 10.03) (Supplementary Table S4). Multivariable logistic regression adjusted for age and sex was done excluding G/G genotype. The results remained consistent with G/A even without inclusion of G/G genotype, confirms its independent contribution to RA risk within the Indian population (Supplementary Table S5).

**FIGURE 4:**
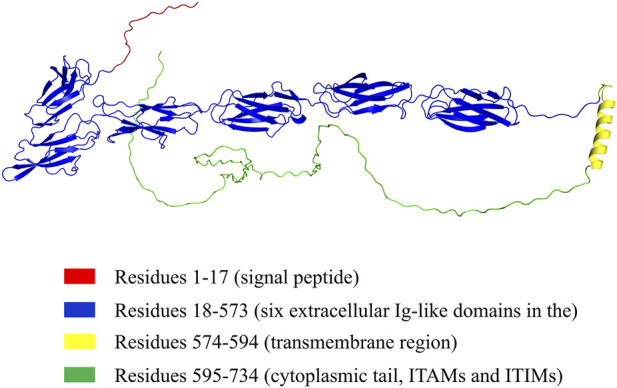
Predicted tertiary structure and domain architecture of FCRL3. The 3D model was generated *via* homology modeling (SWISS-MODEL), with individual functional domains labelled using PyMOL software.

**FIGURE 5:**
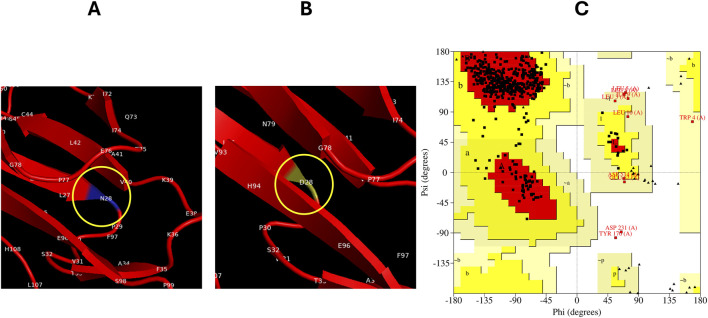
Structural modelling and quality assessment of FCRL3. **(A)** 3D structure of the wild-type FCRL3 protein. **(B)** Modelled structure of the N28D variant highlighting the site of the amino acid substitution. **(C)** Ramachandran plot analysis validating the stereochemical quality of the modeled protein, illustrating the distribution of residues within favoured, allowed, and outlier regions.

Further, to demonstrate the association of non-synonymous SNP rs7522061 with susceptibility of RA, we investigated the potential damaging impact of the N28D amino acid substitution in FCRL3 protein. This amino acid substitution results because of G allele in individuals carrying the rs7522061 G/A genotype, following a dominant model. To evaluate the functional consequence of N28D, we employed a hierarchical computational approach. While sequence-based predictors, including SIFT, Polyphen, and CADD indicated subtle effect of N28D on FCRL3 protein structure (SIFT = 0.691, Polyphen = 0.0 and CADD = 2.7), these tools often fail to capture the complexities of allosteric modulation and misclassify the gain-of-function variations as benign or tolerated. Consequently, analysis *via* DynaMut2 revealed that the N28D substitution exerts stabilizing effect on the FCRL3 protein scaffold with ΔΔG of 0.23 kcal/mol compared to wild type. This gain in thermodynamic stability and alterations in conformational flexibility indicates that the N28D functions as an allosteric regulator, propagating structural perturbations across the protein scaffold. This energy landscape shift provides a mechanistic basis for molecular docking studies, as the rigidified variant structure likely alters the binding affinity and interfacial dynamics with the downstream signalling protein SHP-1. Therefore, we proceeded for comparative molecular docking and dynamics simulations. The strategy was designed to elucidate how the N28D-induced conformational changes disturb the downstream inhibitory signalling interface between wild type and FCRL3 N28D variant protein with the its signalling partner SHP-1. Due to unavailability of an experimentally determined X-RAY crystallised structure of FCRL3 protein, we obtained the corresponding primary protein sequence of FCRL3 *via* the UNIPROT database (Biasini et al., 2014). The sequence was employed to generate FCRL3 3D protein structure through SWISS-MODEL and the FCRL3 protein’s domains were labelled using PyMOL software (Figure 4). The simulated structure of FCRL3 protein was validated using PROCHECK programs. The Ramachandran plot analysis of the modelled FCRL3 protein (Figure 5C) indicated that 88.9% of residues were situated within the most favourable regions. Furthermore, 8.1% and 1.7% of the residues were distributed within additionally and generously allowed regions respectively. The integrity of the model was subsequently verified using additional parameters such as main-chain bond lengths and Chi1/Chi2 plots, both metrics confirmed that the model adheres to standard stereochemical limits. For the variant FCRL3 protein, N28D was inserted at 28th amino acid position (asparagine to aspartic acid) using PyMOL software (Figures 5A,B), SHP-1 protein structure was downloaded from PDB. Molecular docking was performed between FCRL3 protein (wild type and N28D variant) and SHP-1 using HADDOCK server to predict their interaction. The docking results showed that the FCRL3 N28D protein forms a highly stable structural complex with SHP-1, with a HADDOCK score of −59.8 ± 16.4 compared to the wild-type FCRL3 complex (−52.7 ± 8.6) HADDOCK score (−59.8 ± 16.4 vs. −52.7 ± 8.6). Also other parameters, such as Van der Waals and electrostatic energies, and a larger buried surface area exhibited better scores for FCRL3 N28D -SHP-1 complex. This indicates that the N28D variant protein enhances its binding affinity with SHP-1, even though the statistical separation from other clusters (Z-score) remained comparable (Table 5).

**TABLE 5:** Docking scores comparison between FCRL3 wild type vs. N28D variant binding strength with SHP-1.

Interaction energies	Wild type FCRL3-SHP-1			FCRL3 N28D -SHP-1		
Cluster rank	Cluster 5	Cluster 8	Mean	Cluster 9	Cluster 2	Mean
HADDOCK score	−52.7 ± 8.6	−54.2 ± 7.9	−53.45 ± 5.84	−59.8 ± 16.4	−62.4 ± 14.8	−61.1 ± 11.0
Van der waals energy	−22.0 ± 6.8	−24.8 ± 6.2	−23.40 ± 4.60	−31.7 ± 8.0	−34.5 ± 7.9	−33.1 ± 5.6
Electrostatic energy	−112.5 ± 12.4	−128.6 ± 11.5	−120 ± 8.46	−133.2 ± 46.0	−141.8 ± 38.5	−137.5 ± 30.0
Desolvation energy	−8.2 ± 1.6	−7.5 ± 2.1	−7.85 ± 1.32	−1.6 ± 5.6	−2.8 ± 6.1	−2.2 ± 4.1
Restraints violation energy	0.9 ± 0.3	1.1 ± 0.4	1.00 ± 0.25	0.5 ± 0.6	0.4 ± 0.5	0.5 ± 0.4
Buried surface area	1,008.1 ± 175.8	1,024 ± 162	1,016.05 ± 119.53	1,286.2 ± 319.4	1,352.6 ± 298.7	1,319.4 ± 218.7
Z- score	−1.3	−1.4	−1.35	−1.3	−1.6	−1.5
RMSD from overall lowest energy structure	11.8 ± 0.3	12.3 ± 0.4	12.05 ± 0.25	15.3 ± 0.3	14.7 ± 0.4	15.0 ± 0.2

**FIGURE 6:**
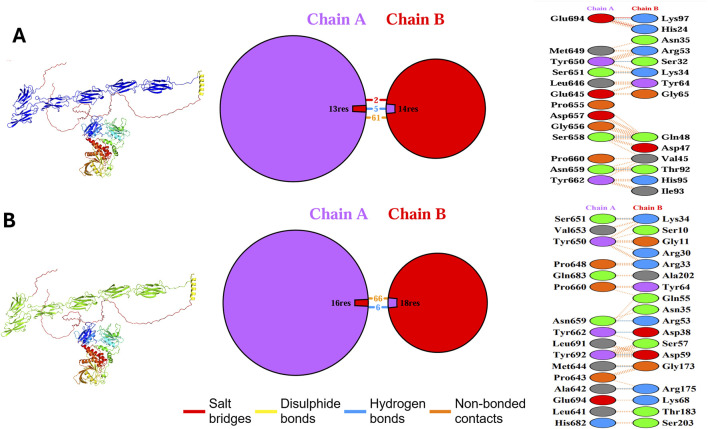
*In-silico* analysis of the FCRL3–SHP-1 binding interface. Interaction diagrams generated *via* PDBsum illustrate the residue-level contacts between the FCRL3 (Chain A) and the SHP-1 phosphatase (Chain B). Comparisons between **(A)** the Wild-type complex and **(B)** the N28D variant complex reveal the network of hydrogen bonds, salt bridges, and non-bonded interactions.

The binding interface residues of the wild-type and FCRL3 N28D -SHP-1 complexes were analysed using PDBsum. As shown in Figure 6B, the FCRL3 N28D-SHP-1 complex formed six hydrogen bonds involving the following residues: SER651/LYS34, ASN659/ARG53, TYR662/ASP38, TYR692/ASP59, MET644/GLY173, and ALA642/ARG175. In addition, 66 other non-bonded interactions were observed. In contrast, the wild-type FCRL3-SHP-1 complex exhibited five hydrogen bonds (GLU694/LYS97, TYR650/SER32, SER651/LYS34, SER658/GLN48, and ASN659/THR92) and 61 non-bonded interactions (Figure 6A). The residue GLU694 in the wild-type complex also formed salt bridges with LYS97 and HIS24. These interaction analyses, together with the HADDOCK docking scores, indicate that the N28D variant forms a more extensive interaction interface with SHP-1 compared to the wild-type protein.

### 3.2 Root mean square deviation (RMSD)

**FIGURE 7:**
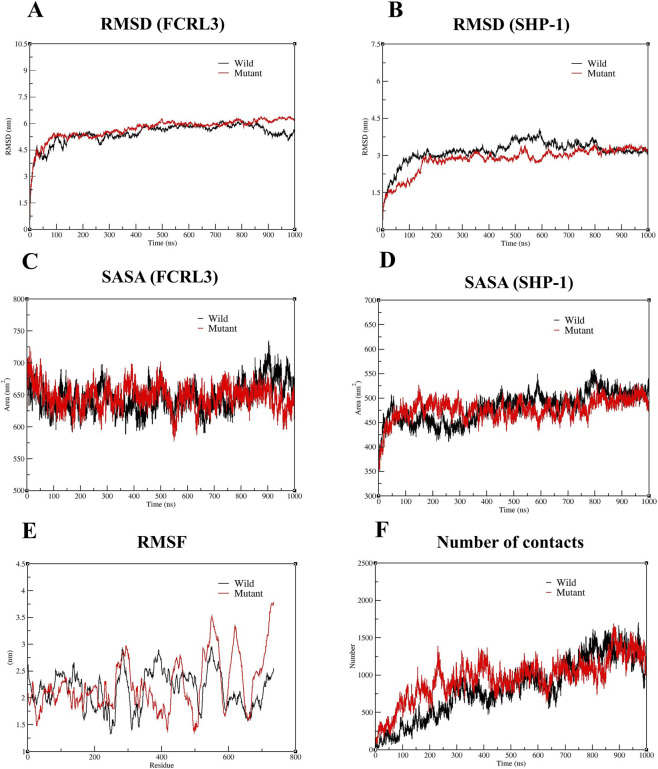
CG-MD simulation results for 1,000 ns trajectories. Post-simulation analysis was performed for wild-type and variant complexes. **(A)** RMSD for FCRL3. **(B)** RMSD for SHP-1. **(C)** SASA for FCRL3. **(D)** SASA for SHP-1. **(E)** RMSF. **(F)** Number of contacts formed over time.

The structural stability and conformational convergence of both FCRL3 and SHP-1 were evaluated using RMSD analysis throughout the 1,000 ns stimulation trajectory. (Figure 7A), Both wild-type and variant FCRL3 systems initially equilibrated within the first 100 ns, reaching a plateau between 5.0 and 6.0 nm. However, the wild-type showed a notable deviation dip after 850 ns, whereas the variant maintained a more consistent, slightly higher RMSD profile across the 1,000 ns trajectory. Similarly for SHP-1 (Figure 7B), the overall RMSD values were lower, stabilizing around 3.0–3.2 nm, indicating a more rigid structural core within the complex. While the FCRL3 wild-type SHP-1 displayed significant conformational fluctuations between 450 ns and 650 ns peaking near 4.0 nm, the FCRL3 variant SHP-1 followed a smoother trajectory with fewer stochastic jumps, suggesting that the variation may enhance the local backbone stability of SHP-1. Ultimately, the achievement of steady-state plateaus in both systems confirms that the 1,000 ns coarse-grained simulation provided sufficient sampling for the complex to reach thermodynamic equilibrium, with the observed deviations falling within the expected range for the MARTINI force field.

### 3.3 Solvent accessible surface area (SASA)

SASA was analysed across the 1,000 ns trajectory to assess the surface exposure and global compactness of FCRL3 and SHP-1 proteins. Both FCRL3 wild-type and variant systems exhibited significant but comparable fluctuations, maintaining an average SASA of approximately 650 nm^2^ (Figure 7C). The high degree of overlap between the wild type and N28D suggests that the variation does not induce major structural expansions or contractions in FCRL3. Both forms preserving a similar degree of hydrophobic burial. Conversely, the SHP-1 (Figure 7D) SASA profiles stabilized between 450 and 520 nm^2^ following an initial 100 ns equilibration phase. The FCRL3 N28D -SHP-1 complex demonstrated a remarkably stable surface area throughout the simulation, whereas the wild-type exhibited notable dips in exposure particularly between 200 and 400 ns before rising again toward the end of the trajectory. This suggests that the SHP-1 with variant maintains a slightly more consistent than the wild-type. Overall, the consistent steady SASA values observed for both complexes across the 1,000 ns timescale confirm that the FCRL3 and SHP-1 proteins remain well-folded and structurally intact in both their wild-type and variant states.

### 3.4 Root mean square fluctuation (RMSF)

To investigate local flexibility and regional dynamics at a residue-level resolution, RMSF was calculated for both the FCRL3 wild-type and variant across the 1,000 ns trajectory (Figure 7E). In both the systems, first 500 residues exhibited highly similar fluctuation patterns, with baseline values ranging between 1.5 and 3.0 nm. This suggests that the N-terminal and central domains maintain comparable structural stability in both systems. However, a significant divergence in conformational mobility emerged in the C-terminal region (residues 500–800). The variant displayed markedly increased flexibility, characterized by sharp peaks at residues 550 and 620, followed by a substantial surge in fluctuation at the C-terminus, which reached nearly 3.8 nm. In contrast, the wild-type remained relatively stable in these regions, with fluctuations largely contained below 2.5 nm. These results suggest that, although the global fold is preserved, the variant triggers localized destabilization and increases structural plasticity within the C-terminal domain, which may influence the overall binding affinity and the long-range interaction dynamics of the protein complex.

### 3.5 Binding interface contact dynamics

To quantify the binding intensity and interface formation over time, the inter-protein number of contacts was calculated throughout the 1,000 ns trajectory (Figure 7F). The analysis reveals a distinct difference in the initial binding kinetics: the N28D complex exhibited a rapid increase in contact formation within the first 250 ns, quickly reaching a plateau of approximately 1,000 contacts. In contrast, the wild-type complex showed a gradual progression, maintaining fewer contacts (between 500 and 800) during the first half of the simulation. However, beyond 600 ns mark, the wild-type experienced a significant rise in interfacial interactions, eventually converging with the variant at a steady state of 1,200–1,500 contacts for the remaining simulation. These results suggest that while both systems eventually achieve a similar level of binding saturation, the variant facilitates a more rapid establishment of the protein-protein interface, indicating an enhanced initial recruitment or docking efficiency compared to the wild-type.

### 3.6 Radius of gyration (rg)

**FIGURE 8:**
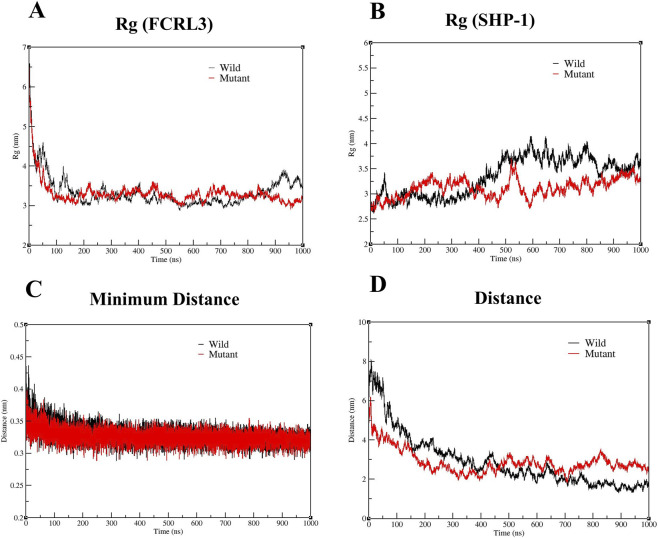
Global compactness and inter-protein distance analysis of the FCRL3–SHP-1 complex. **(A,B)** Rg illustrating the global compactness and structural integrity of the complex over 1000 ns. **(C)** Minimum distance between the FCRL3 and SHP-1 interfaces. **(D)** Average inter-protein distance calculated across the simulation trajectory.

The global compactness and structural integrity of the protein-protein complex were evaluated through Rg analysis over the 1,000 ns trajectory. Both wild-type and variant FCRL3 systems exhibited rapid initial compaction, with Rg values dropping from approximately 6.0 nm to a stable plateau between 3.0 and 3.5 nm within the first 100 ns (Figure 8A). While the variant maintained consistent level of compactness for the remainder of the simulation, the wild-type showed a slight expansion toward 3.8 nm in the final 100 ns, suggesting a minor conformational relaxation. In contrast, SHP-1 (Figure 8B) revealed a distinct divergence in structural behaviour: the wild-type -SHP-1 exhibited a progressive increase in Rg, reaching peaks above 4.0 nm between 600 ns and 800 ns, indicating a loss of compactness or significant conformational expansion. Conversely, the SHP-1 variant remained significantly more stable and compact, oscillating steadily around 3.0–3.3 nm. These result indicate that although FCRL3 retains its folded state in both systems, the variant appears to exert a stabilizing effect on the global compactness of SHP-1 within the complex, thereby mitigating the structural expansions observed in the wild-type trajectory.

### 3.7 Interfacial distance

To assess the proximity and binding persistence of protein-protein interface, the minimum distance between FCRL3 and SHP-1 was calculated throughout the 1,000 ns trajectory (Figure 8C). Throughout the 1,000 ns trajectory, the N28D complex demonstrated remarkable structural stability, maintaining a tight and consistent inter-protein distance of approximately 0.15 nm from the early equilibration phase. This lack of fluctuation suggests a highly stable binding orientation. In contrast, the wild-type complex exhibited significant conformational instability during the mid-stages of the simulation. While the complex initially maintained close proximity, a prominent peak emerged between 500 ns and 650 ns, where the minimum distance nearly doubled to 0.30 nm, indicating a transient widening or partial relaxation of the binding interface during this interval. However, beyond 700 ns mark, the wild-type distance decreased and stabilized near 0.18 nm, ultimately converging towards N28D profile. These findings suggest that while both systems eventually achieve a close-contact equilibrium, the N28D variant confers enhanced interfacial rigidity and persistent proximity, potentially facilitating more efficient downstream inhibitory signalling compared to the more flexible wild-type interaction.

### 3.8 Centre-of-mass distance

The stability of the FCRL3–SHP-1 interaction interface was evaluated by monitoring the center of mass distance between the two proteins throughout the 1,000 ns CG- MD simulation (Figure 8D). Both complexes showed a rapid decrease in inter-protein distance during the initial equilibration phase (first 150 ns). Thereafter, the FCRL3 N28D–SHP-1 complex maintained a more constant and stable distance, fluctuating narrowly between 2.5 and 3.0 nm for the majority of the simulation trajectory. In contrast, the wild-type FCRL3–SHP-1 complex displayed greater variability, reaching a closer average distance (1.5–2.0 nm) at the end but with more pronounced fluctuations even after 600 ns? These results indicate that the N28D variation promotes a constant and stable protein-protein interface distance throughout the simulation, suggesting enhanced binding stability compared to the wild-type complex.

### 3.9 RMS deviation and cluster index

**FIGURE 9:**
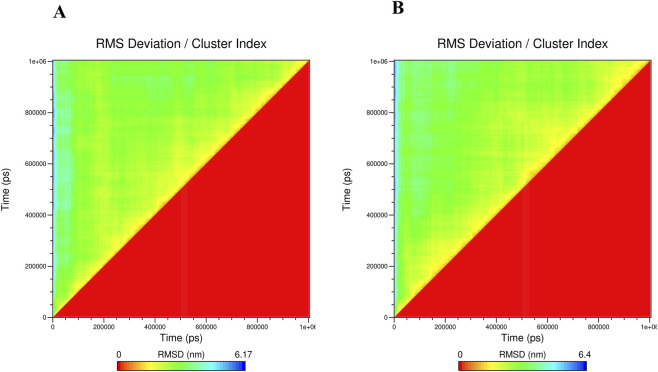
RMS deviation and Cluster Index analysis for FCRL3-SHP-1 Complexes. **(A)** Wild FCRL3-SHP-1. This graph presents the RMS deviation (RMSD, red line, left y-axis) and the cluster index (blue line, right y-axis) over the 1,000 ns CG-MD simulation trajectory. **(B)** N28D FCRL3-SHP-1. This graph shows the corresponding RMSD and cluster index for the N28D variant complex.

The RMS deviation profiles were analysed to assess conformational stability throughout the 1,000 ns CG-MD trajectory. The wild-type complex (Figure 9A) exhibited moderate fluctuations during the initial 200–300 ns, with RMS deviation values ranging between 0.4 and 0.7 nm, followed by gradual stabilization with a mean RMS deviation of approximately 0.50 nm, although intermittent deviations persisted throughout the simulation, suggesting residual flexibility. In contrast, the N28D variant complex (Figure 9B) reached a stable plateau more rapidly (within 150 ns) and maintained a consistent lower RMS deviation (mean ∼0.40 nm, range 0.35–0.45 nm) with minimal oscillations over the remaining 850 ns? Trajectory clustering analysis further supported these observations: the wild-type sampled a wider distribution of conformational substates, whereas the N28D complex converged to a single dominant cluster, indicating enhanced structural homogeneity. Collectively, these results indicate that the N28D substitution is associated with greater conformational stability in the simulated environment of the FCRL3–SHP-1 interaction interface compared to the wild-type protein. Overall, the molecular docking and CG-MD simulations suggested that the N28D variant may form a more stable complex with SHP-1 compared to the wild-type protein, which could possibly exert negative signalling to the Tregs and cause the dysregulation, a hallmark of RA.

## 4 Discussion

FCRL3, a transmembrane immunoregulatory receptor mainly expressed on Tregs, plays a vital role in maintaining peripheral immune tolerance. Its immune-regulatory role depends on its intracellular domains, namely, ITAM and ITIM. Specifically, upon phosphorylation, the ITIM residues (Tyr650, Tyr662, Tyr692, and Tyr722) recruit the phosphatases SHP-1 and SHP-2, which then attenuate TCR signaling in Tregs by dephosphorylating key signaling molecules such as LCK and ZAP-70. Both (LCK and ZAP70) function as a key enzymatic driver within the proximal signalling cascade of the TCR-CD3 complex in Treg. The TCR-CD3 complex starts the signalling process upon ligand binding, which is facilitated by LCK through phosphorylating the ζ-chains of ITAM located within the CD3 cytoplasmic tails (Chan et al., 2016). The phosphorylation ITAM creates high affinity docking site for ZAP70, facilitating its recruitment, activation, and the subsequent amplification of downstream signalling in Tregs. This cascade triggers the activation of phospholipase-C and other downstream events, finally triggers the activation of nuclear factor of activated T-cells (NFAT) and its concurrent nuclear entry (Fernández-Aguilar et al., 2023; Sana et al., 2021; Maruyama et al., 2012; Muzammil and Devi, 2026). In the nucleus, NFAT initiates the transcription of forkhead box protein 3 (FOXP3) gene. FOXP3 is recognized as a master transcription factor governing the functional program of the Treg lineage. It functions by driving the expression of anti-inflammatory mediators, specifically IL-35, IL-10, and TGF-β (Wu et al., 2006b; Gravano and Vignali, 2012). However, this regulatory process is highly influenced by FCRL3, by attenuating the TCR-CD3 signalling pathway through SHP-1 within these cells (Kochi et al., 2009). Any genetic variation that enhances the binding between phosphorylated FCRL3 ITIM and SHP-1 may potentiate inhibitory signalling within Tregs. This interaction relieves the autoinhibition of SHP-1, fully activating its phosphatase domain, Once activated SHP-1 dephosphorylates key TCR signalling molecules, including LCK at Tyr-394 and ZAP-70 at Tyr-493, to suppress signalling. Consequently, downstream TCR signalling is attenuated, leading to the modulation and potential impairment of Treg suppressive function. This disruption may compromise peripheral tolerance, thereby promoting an environment conducive to autoimmunity (Kochi et al., 2009; Lorenz, 2008; Wang et al., 2012; Craggs and Kellie, 2001; Xu et al., 2002). The genotypic analysis of 226 R A patients and 239 control samples demonstrated that the missense SNP, rs7522061 G/A genotype was significantly associated with an increased risk of RA (unadjusted OR = 1.84, 95% CI = 1.21–2.79, *p =* 0.020 after Benjamini-Hochberg correction). To verify the reliability of this association, a multivariable binary logistic regression analysis was performed. After adjusting age and sex, the G/A genotype remained strongly associated with RA risk under the genotypic model, with an increased adjusted OR of 2.03 (95% CI = 1.27–3.23, *p =* 0.0028) whereas the other SNPs rs11264799, rs11264797 (monomorphic), and rs7549100, showed no significant association in the initial unadjusted analysis. Overall, the pooled analysis confirmed that the observed association between rs7522061 G/A and RA susceptibility is consistent across the cohort and independent of gender. The rs7522061 G/A variant, induces an asparagine-to-aspartic amino acid change at the nucleotide level, the ancestral allele (A) codes for asparagine (AAC or AAT codon), whereas the variant allele (G) changes the codon to GAC or GAT, which codes for aspartic acid. Therefore, individuals with the heterozygous G/A genotype have one normal allele (encoding asparagine, N) and one variant allele (encoding aspartic acid, D) at amino acid position 28. This results in the N28D amino acid substitution in the extracellular Ig-like domain of the FCRL3 protein. To evaluate the impact of the rs7522061 G/A polymorphism on RA susceptibility, an *in silico* analysis was performed. Due to the absence of an experimental FCRL3 crystal structure, we generated a three-dimensional model using the AlphaFold database *via* SWISS-MODEL. The N28D variation was introduced using PyMOL, and Molecular docking of the FCRL3–SHP-1 (PDB ID: 3PS5) complexes was performed in duplicate using the HADDOCK 2.4 web server. To ensure the reliability of the predicted binding poses, the mean scoring values of wild-type and N28D variant complexes were calculated and utilized for subsequent analysis. The FCRL3 N28D–SHP-1 complex yielded a superior HADDOCK score (mean of top two clusters: 61.1 ± 11.0) compared to the wild-type complex (mean of top two clusters: 53.45 ± 5.84), along with more favourable van der Waals (-33.1 vs. - 23.40), electrostatic (-137.5 vs. - 120), and buried surface area (1,319.4 vs. 1,016.05 Å^2^) parameters, indicating enhanced binding affinity. To evaluate dynamic stability over an extended timescale, the CG-MD simulation was run for 1,000 ns using GROMACS with the MARTINI force field. CG-MD simplifies protein representation by grouping heavy atoms into interaction beads, allowing microsecond-scale simulations that are computationally inaccessible with all-atom models, while maintaining reasonable accuracy for large protein-protein complexes. Throughout 1,000 ns trajectory, the N28D complex exhibited more stable RMSD for SHP-1, a tighter and more consistent minimum distance (0.15 nm) between the proteins, a greater number of inter-protein contacts (rapidly reaching 1,000 contacts and plateauing at 1,200–1,500), and a more stable Rg for SHP-1, indicating enhanced compactness and interfacial rigidity compared to the wild-type. Collectively, these computational findings are consistent with the hypothesis that the N28D variant may allosterically stabilize the FCRL3–SHP-1 interaction where the extracellular N28D substitution possibly induces a conformational shift that is transmitted across the transmembrane domain. This shift likely modulates the accessibility or phosphorylation status of intracellular ITIM. Optimizing the SHP-1 docking interface may enhance the inhibitory signalling within Tregs, thereby reducing immunosuppressive capacity and compromising peripheral tolerance a process implicated in RA pathogenesis. However, several limitations are acknowledged: the FCRL3 model was derived from AlphaFold with high uncertainty in the transmembrane and cytoplasmic regions due to poor template coverage and intrinsic disorder; the CG-MD simulations were performed in aqueous solution without an explicit membrane environment, which may affect the orientation of the transmembrane helix and cytoplasmic tail; and the observed enhanced interaction between ITIM -SHP-1 based on hypothesis-generated which requires experimental validation through co-immunoprecipitation or functional Treg suppression assays. Nevertheless, this study establishes rs7522061 as a significant RA susceptibility variant in the Indian population and provides a plausible structural mechanism by which the N28D variant may augment FCRL3–SHP-1 signalling, offering a foundation for future therapeutic target for autoimmune diseases.

## 5 Conclusion

This study represents the first integrated analysis of *FCRL3* SNPs rs7522061, rs11264799, rs11264797, and rs7549100 within an Indian cohort. Among these variants screened, the rs7522061 genotype (G/A) was significantly linked with RA risk. The results also confirm that the *FCRL3* SNP rs7522061 locus contributes to the genetic architecture of RA in this demographic, showing no evidence of gender-specific modulation. In addition to genetic findings, the *in silico* results suggests that the FCRL3 N28D substitution impacts structural stability and compactness of protein, which influence SHP-1 recruitment. This mechanism may enhance the inhibitory signalling within Tregs, thereby diminishing their immunosuppressive capacity and compromising peripheral tolerance a process implicated in RA pathogenesis.

## Data Availability

The original contributions presented in the study are included in the article/Supplementary Material, further inquiries can be directed to the corresponding author.

## Supplementary material

SUPPLEMENTARY TABLES S1 PCR and qPCR conditions to amplify SNP target regions in *FCRL3* gene.

SUPPLEMENTARY TABLE S2 Participant enrolment and genotyping flow (STREGA compliant).

SUPPLEMENTARY TABLE S3 STREGA quality control and genotyping summary.

SUPPLEMENTARY TABLE S4 Genotype distribution of FCRL3 rs7522061 and unadjusted association with RA risk excluding GG genotype (n = 275).

SUPPLEMENTARY TABLE S5 Multivariable binary logistic regression analysis of rs7522061 genotypes and RA risk excluding GG genotype (Genotypic model, AA as reference).

SUPPLEMENTARY TABLE S6 Distribution of FCRL3 genotypes and their association with RA risk among male and female subgroups in the Indian population.
