# Supplementary material for: Association of FCRL3 gene variants with rheumatoid arthritis susceptibility in the indian population: a combined case-control and in- silico analysis
Source: Front Bioinform. 2026 Jun 3;6:1809854. doi: 10.3389/fbinf.2026.1809854 (PMC13273044; doi:10.3389/fbinf.2026.1809854)
Supplement: Supplementary file 1 [file Supplementaryfile1.docx]

#load libraries

library(dplyr)

library(readxl)

library(stats)

#load file

file <- read_xlsx("path/to/file.xlsx")

# Convert to factor with correct biological labels

file$Genotype <- factor(

file$Genotype_numerical,

levels = c(0,1,2),

labels = c("AA","GA","GG")

)

model_factor <- glm(RA ~ Genotype + Age + Sex,

data = file,

family = binomial)

summary(model_factor)

coef_table <- summary(model_factor)$coefficients

wald <- (coef_table[, "Estimate"] / coef_table[, "Std. Error"])^2

OR <- exp(coef(model_factor))

CI <- exp(confint(model_factor))

final_table <- data.frame(

Variable = rownames(coef_table),

B = coef_table[, "Estimate"],

SE = coef_table[, "Std. Error"],

Wald = wald,

df = 1,

OR = OR,

CI_lower = CI[,1],

CI_upper = CI[,2],

p = coef_table[, "Pr(>|z|)"]

)

final_table
