# Supplementary material for: Association of FCRL3 gene variants with rheumatoid arthritis susceptibility in the indian population: a combined case-control and in- silico analysis
Source: Front Bioinform. 2026 Jun 3;6:1809854. doi: 10.3389/fbinf.2026.1809854 (PMC13273044; doi:10.3389/fbinf.2026.1809854)
Supplement: Supplementary file 2 [file Table1.docx]

**Supplementary Table 1**

PCR conditions to amplify the FCRL3 SNP target regions and genotyping

| **A. PCR conditions to amplify SNP target regions within FCRL3 gene** | | | | | | |
| --- | --- | --- | --- | --- | --- | --- |
| SNP ID/ amplification product size (bp) | Initial Denaturation | Denaturation | Annealing | Extension | Final Extension | No. of Cycles |
| rs7522061/  441bp | 98°C/1min | 98°C/10 sec | 60°C/5 sec | 72°C/30 sec | 72°C/5 min | 40 |
| rs11264799/  465bp |  |  | 56°C/8 sec |  |  |  |
| rs11264797/  502bp |  |  | 58°C/5 sec |  |  |  |
| rs7549100/  480bp |  |  | 60°C/7 sec |  |  |  |
| **B. qPCR conditions for genotyping FCRL3 SNPs by HRMA** | | | | | | |
| SNP ID | Initial Denaturation | Denaturation | Annealing | Melting curve  (1sec Temperature intervals/ Time) | | No. of Cycles |
| rs7522061 | 95°C/5 min | 95°C/15 sec | 60°C/40 sec | 65°C to 85°C (5 sec) | | 45 |
| rs11264799 |  |  | 56°C/40 sec |  |  |  |
| rs11264797 |  |  | 58°C/40 sec |  |  |  |
| rs7549100 |  |  | 60°C/40 sec |  |  |  |

**Supplementary Table 2.**

Participant enrolment and genotyping flow (STREGA compliant)

| **Stage** | **RA Cases** | **Healthy Controls** | **Total** |
| --- | --- | --- | --- |
| **Initially approached / screened** | 235 | 245 | 480 |
| **Excluded (with reasons)** | 9 | 6 | 15 |
| **Did not meet 2010 ACR/EULAR RA criteria** | 5 | - | 5 |
| **Final enrolled participants** | 226 | 239 | 465 |
| **DNA successfully extracted** | 226 (100%) | 239 (100%) | 465 (100%) |
| **Genotyping attempted for SNPs (rs7522061, rs11264799, rs11264797 rs7549100)** | 226 | 239 | 465 |
| **Successfully genotyped SNP rs7528684** | 226 (100%) | 239 (100%) | 465 (100%) |
| **Final analysed** | 226 | 239 | 465 |

**Supplementary Table 3.**

STREGA quality control and genotyping summary

| **STREGA Item** | **Parameter applied in this Study** |
| --- | --- |
| **Call Rate (Per SNP)** | 100% for FCRL3 SNPs rs7522061, rs11264799, rs11264797, rs7549100 |
| **Missingness (by Case/Control)** | 0% missingness. Genotype data was completed for all 226 RA cases and 239 controls. No samples excluded due to genotyping failure. |
| **Differential missingness** | Not applicable (zero missing genotypes in both groups) |
| **Genotyping error rate** | < 0.1% (estimated from technical duplicates) |
| **Duplicate concordance** | Technical duplicates were performed for every sample (n=465) with 100% concordance. |
| **Sanger sequencing validation** | 10% of samples from ambiguous HRMA melt curves were validated by Sanger sequencing, samples were independently confirmed (100% concordance). |
| **Blinded Genotyping** | Laboratory personnel were blinded to case/control status during genotype assignment. |
| **Hardy-Weinberg Equilibrium (Controls)** | rs7522061: *p* = 0.0613  rs11264799: *p* = 0.4760  rs7549100: *p* = 0.3739 |
| **rs11264797** | Monomorphic (only G/G genotype observed in entire cohort) |
| **Population Stratification Handling** | Population stratification handling- Recruitment of sample from same hospital (patients from across India)  - Self-reported ethnicity  - Adjustment for age and sex in regression model (only for significant SNP rs7522061)  - Careful matching during enrolment |
| **Multiple Testing Correction** | Benjamini-Hochberg False Discovery Rate (FDR) correction applied across the tested genotypes. |
| **Covariate adjustment** | Age and sex included as covariates in multivariable logistic regression |

**Supplementary Table 4.**

Genotype distribution of FCRL3 rs7522061 and unadjusted association with rheumatoid arthritis risk excluding G/G genotype (n = 275)

| **SNP ID** | **Genotypes** | ***Controls***  ***n (%)*** | ***RA***  ***n (%)*** | ***χ^2^*** | **OR** | **95% C. I** | **Z-stats** | ***p-value*** |
| --- | --- | --- | --- | --- | --- | --- | --- | --- |
| **Rs7522061** | A/A  (ref) | 181 (78.4%) | 136 (64.8%) | - | 1.00 | - | - | - |
|  | **G/A** | **50 (21.6%)** | **74 (35.2%)** | **10.03** | **1.96** | **1.29-3.00** | **3.147** | **0.0015** |

**Supplementary Table 5.**

Multivariable binary logistic regression analysis of rs7522061 genotypes and RA risk excluding G/G genotype (Genotypic model, A/A as reference).

| **Variable** | **B (coeff)** | **SE** | **Wald (z2)** | **Adjausted OR** | **95% CI** | ***p-value*** |
| --- | --- | --- | --- | --- | --- | --- |
| A/A (Reference) | - | - | - | 1.00 | - | - |
| G/A | 0.7138 | 0.238 | 3.002 | 2.04 | 1.28 - 3.25 | 0.0027 |
| Sex (male vs female) | -0.3046 | 0.216 | -1.411 | 0.738 | 0.483 - 1.126 | 0.158 |
| Age (per year) | 0.0864 | 0.011 | 7.930 | 1.090 | 1.06 - 1.11 | 0.0001 |

.

**Supplementary Table 6.**

| **Male-stratified association analysis of FCRL3 SNP genotypes with RA susceptibility** | | | | | | | | | | |
| --- | --- | --- | --- | --- | --- | --- | --- | --- | --- | --- |
| SNP ID | **Genotypes** | ***Controls***  ***n (%)*** | ***RA***  ***n (%)*** | ***χ^2^*** | **OR** | **95% C. I** | **Z-stats** | ***Original p-value*** | ***Adj***  ***p-value*** | ***FDR%*** |
| rs7522061 | A/A  (ref) | 85 (79.4) | 55 (67.1) | - | 1.0 | - | - |  | - | - |
|  | G/A | 18 (16.8) | 23 (28.0) | 3.4260 | 1.895 | 0.94 - 3.81 | 1.795 | 0.0642 | 0.3210 | 0.1605 |
|  | G/G | 4 (3.8) | 4 (4.9) | 0.1480 | 3.814 | 0.89 - 16.2 | 1.811 | 0.7005 | 1.0000 | 0.7005 |
| rs11264799 | G/G  (ref) | 80 (74.8) | 49 (59.8) | - | 1.0 | - | - | - | - | - |
|  | G/A | 24 (22.4) | 29 (35.3) | 3.8295 | 1.892 | 0.99 - 3.59 | 1.949 | 0.0504 | 0.2520 | 0.1605 |
|  | A/A | 3 (2.8) | 4 (4.9) | 0.5571 | 1.777 | 0.38 - 8.17 | 0.739 | 0.4554 | 1.0000 | 0.5692 |
| rs7549100 | T/T  (ref) | 97 (90.7) | 69 (84.1) | - | 1.0 | - | - | - | - | - |
|  | T/C | 10 (9.3) | 13 (15.9) | 1.8298 | 1.827 | 0.75 - 4.40 | 1.343 | 0.1762 | 0.8810 | 0.2936 |
| **Female-stratified association analysis of FCRL3 SNP genotypes with RA susceptibility** | | | | | | | | | | |
| **SNP ID** | **Genotypes** | ***Controls***  ***n (%)*** | ***RA***  ***n (%)*** | ***χ^2^*** | **OR** | **95% C. I** | **Z-stats** | ***Original p-value*** | ***Adj***  ***p-value*** | ***FDR%*** |
| rs7522061 | A/A  (ref) | 96 (72.7) | 81 (56.3) | - | 1.0 | - | - |  | - | - |
|  | G/A | 32 (24.3) | 51 (35.4) | 4.0745 | 1.732 | 1.02 - 2.92 | 2.052 | 0.0435 | 0.2175 | 0.1503 |
|  | G/G | 4 (3) | 12 (8.3) | 3.5337 | 0.341 | 0.10 - 1.08 | 1.821 | 0.0601 | 0.3005 | 0.1503 |
| rs11264799 | G/G  (ref) | 93 (70.5) | 93 (64.6) | - | 1.0 | - | - | - | - | - |
|  | G/A | 35 (26.5) | 46 (31.9) | 1.0517 | 1.300 | 0.77 - 2.19 | 0.949 | 0.3051 | 1.0000 | 0.5085 |
|  | A/A | 4 (3) | 5 (3.5) | 0.0425 | 1.151 | 0.30 - 4.38 | 0.206 | 0.8367 | 1.0000 | 0.8367 |
| rs7549100 | T/T  (ref) | 116 (87.9) | 123 (85.4) | - | 1.0 | - | - | - | - | - |
|  | T/C | 16 (12.1) | 21  (14.6) | 0.3583 | 1.237 | 0.61 - 2.48 | 0.599 | 0.5494 | 1.0000 | 0.6868 |

Distribution of FCRL3 SNP genotypes and their association with RA risk among male and female subgroups in the Indian population.
