# Supplementary material for: Association of FCRL3 gene variants with rheumatoid arthritis susceptibility in the indian population: a combined case-control and in- silico analysis
Source: Front Bioinform. 2026 Jun 3;6:1809854. doi: 10.3389/fbinf.2026.1809854 (PMC13273044; doi:10.3389/fbinf.2026.1809854)

Supplementary figures

All the gel images are original, uncropped gel scans, and not reused for illustrative purposes

Supplementary Figure 1

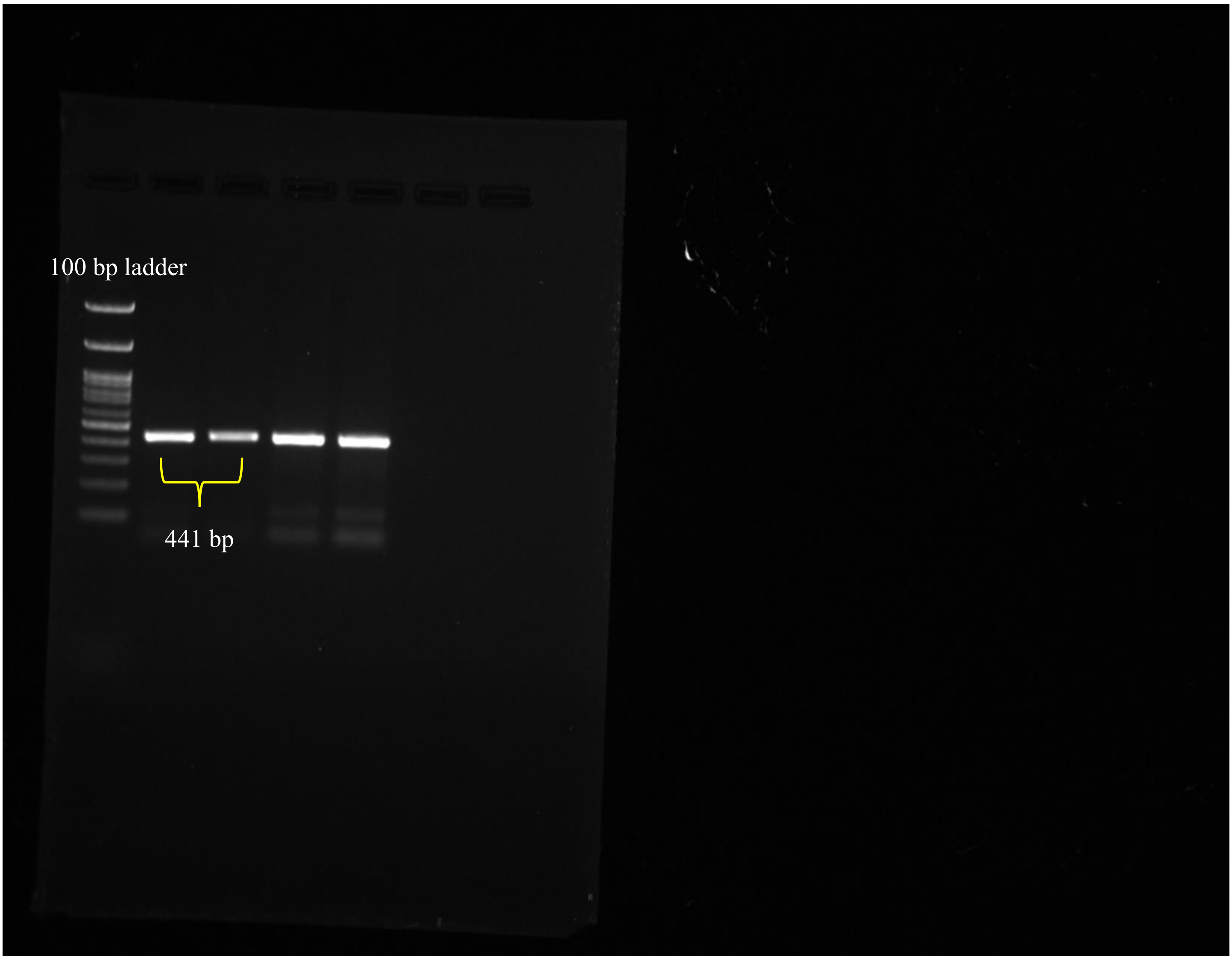

Supplementary Figure 2

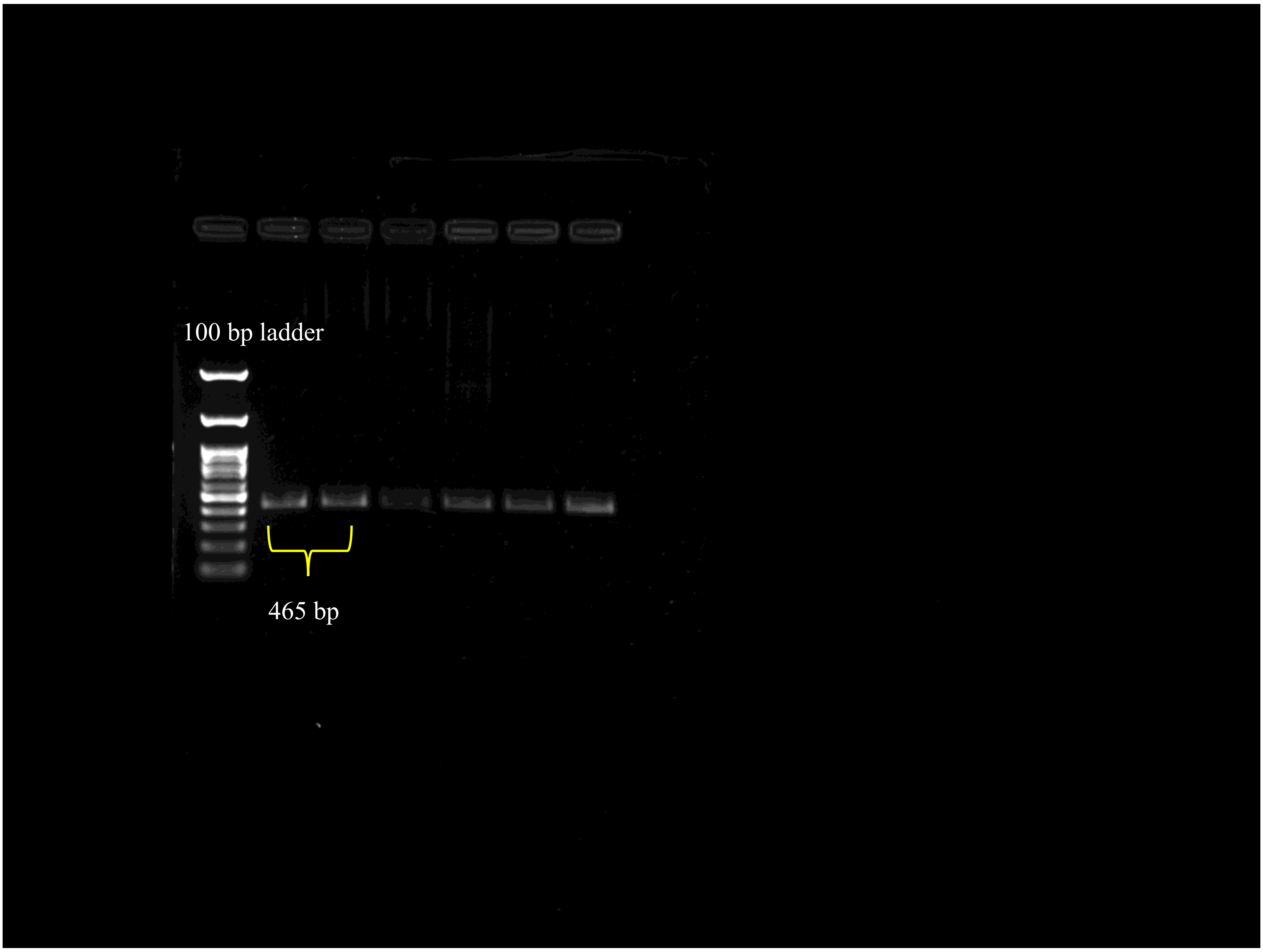

Supplementary Figure 3

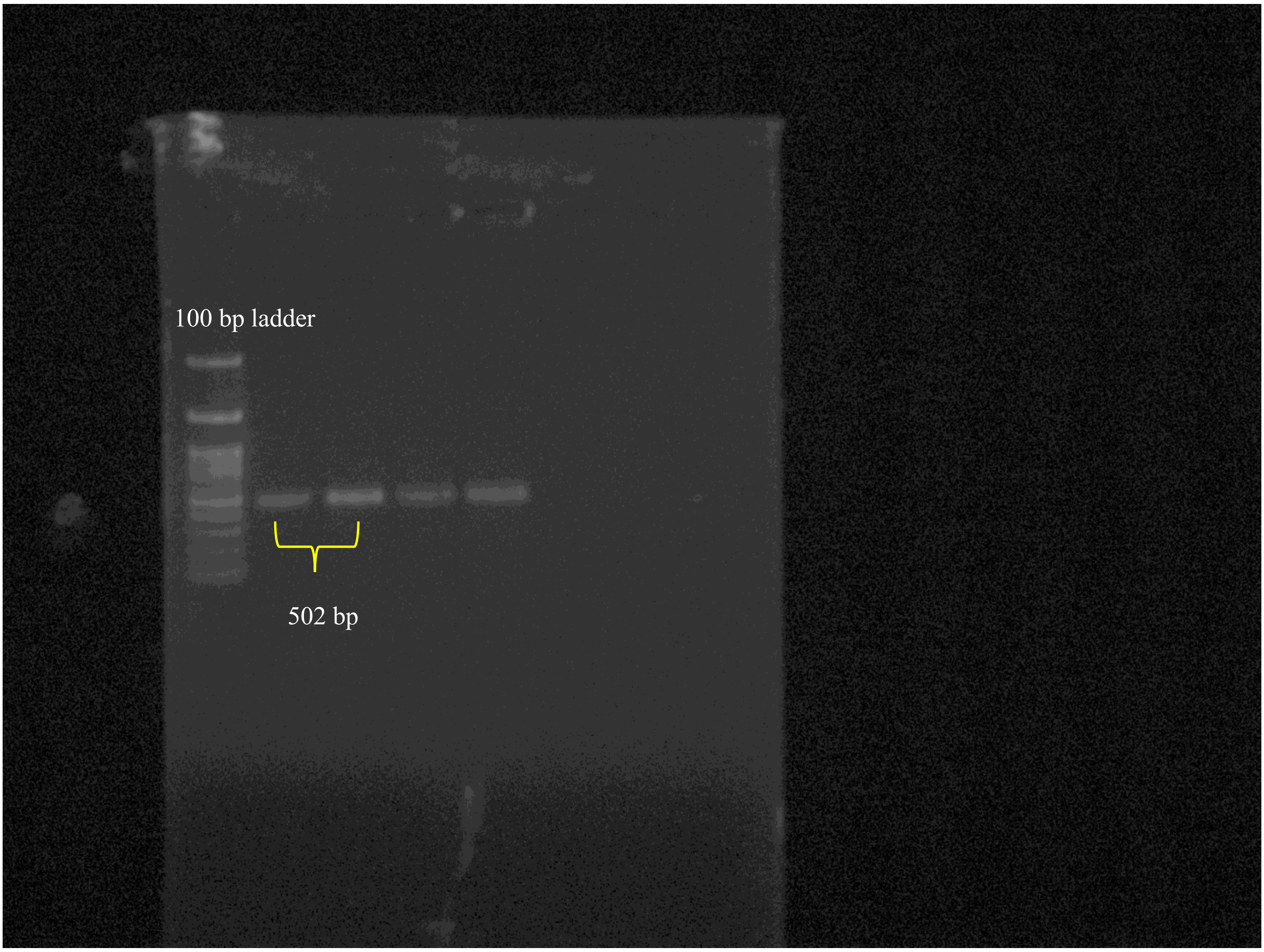

Supplementary Figure 4

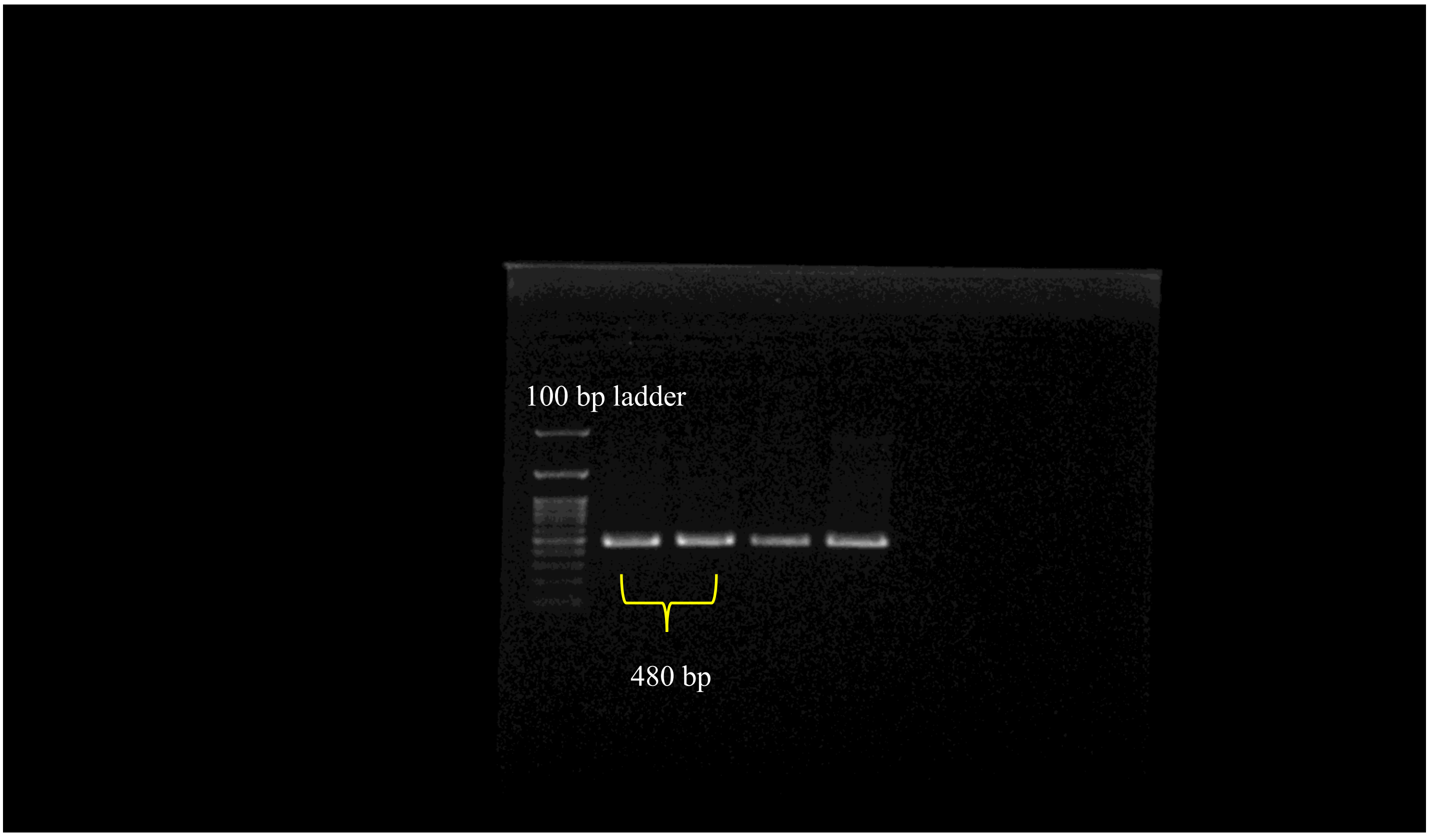

Supplement: Supplementary file 3 [file DataSheet1.pdf]
