# Supplementary material for: Association of FCRL3 gene variants with rheumatoid arthritis susceptibility in the indian population: a combined case-control and in- silico analysis
Source: Front Bioinform. 2026 Jun 3;6:1809854. doi: 10.3389/fbinf.2026.1809854 (PMC13273044; doi:10.3389/fbinf.2026.1809854)
Supplement: Supplementary file 4 [file Supplementaryfile2.docx]

Minimization 01 mdp

define = -DPOSRES -DPOSRES_FC=0 -DNORMANG

integrator = steep

tinit = 0.0

dt = 0.020

nsteps = 3000

nstlog = 100

nstenergy = 100

nstxout-compressed = 1000

compressed-x-precision = 100

cutoff-scheme = Verlet

nstlist = 20

ns_type = grid

pbc = xyz

verlet-buffer-tolerance = 0.005

epsilon_r = 15

coulombtype = reaction-field

rcoulomb = 1.1

vdw_type = cutoff

vdw-modifier = Potential-shift-verlet

rvdw = 1.1

tcoupl = v-rescale

tc-grps = protein solute

tau_t = 1.0 1.0

ref_t = 310.15 310.15

; Pressure coupling:

Pcoupl = C-rescale

Pcoupltype = isotropic

tau_p = 5.0

compressibility = 4.5e-5

ref_p = 1.0

; GENERATE VELOCITIES FOR STARTUP RUN:

gen_vel = yes

gen_temp = 310.15

gen_seed = 7557097424

refcoord_scaling = all

;soft-core-minimization so that single precision GROMACS works here

; Free energy parameters

free-energy = yes

init-lambda = 0.01

sc-alpha = 4

sc-power = 2

sc-coul = yes

nstdhdl = 0

couple-moltype = system

; we are changing both the vdw and the charge. In the initial state, both are on

couple-lambda0 = vdw-q

; in the final state, both are off.

couple-lambda1 = none

couple-intramol = yes

Minimization 02 mdp

define = -DPOSRES -DPOSRES_FC=0

integrator = steep

tinit = 0.0

dt = 0.020

nsteps = 3000

nstlog = 100

nstenergy = 100

nstxout-compressed = 1000

compressed-x-precision = 100

cutoff-scheme = Verlet

nstlist = 20

ns_type = grid

pbc = xyz

verlet-buffer-tolerance = 0.005

epsilon_r = 15

coulombtype = reaction-field

rcoulomb = 1.1

vdw_type = cutoff

vdw-modifier = Potential-shift-verlet

rvdw = 1.1

tcoupl = v-rescale

tc-grps = protein solute

tau_t = 1.0 1.0

ref_t = 310.15 310.15

; Pressure coupling:

Pcoupl = C-rescale

Pcoupltype = isotropic

tau_p = 5.0

compressibility = 4.5e-5

ref_p = 1.0

; GENERATE VELOCITIES FOR STARTUP RUN:

gen_vel = yes

gen_temp = 310.15

gen_seed = 7557097424

refcoord_scaling = all

Equilibration mdp

define = -DPOSRES -DPOSRES_FC=4000

integrator = md

tinit = 0.0

dt = 0.005

nsteps = 50000

lincs_iter = 2

lincs_order = 6

nstlog = 1000

nstenergy = 1000

nstxout-compressed = 1000

compressed-x-precision = 100

cutoff-scheme = Verlet

nstlist = 20

ns_type = grid

pbc = xyz

verlet-buffer-tolerance = 0.005

epsilon_r = 15

coulombtype = reaction-field

rcoulomb = 1.1

vdw_type = cutoff

vdw-modifier = Potential-shift-verlet

rvdw = 1.1

tcoupl = v-rescale

tc-grps = protein solute

tau_t = 1.0 1.0

ref_t = 310.15 310.15

; Pressure coupling:

Pcoupl = berendsen

Pcoupltype = isotropic

tau_p = 5.0

compressibility = 4.5e-5

ref_p = 1.0

; GENERATE VELOCITIES FOR STARTUP RUN:

gen_vel = yes

gen_temp = 310.15

gen_seed = 7557097424

refcoord_scaling = com

Production mdp

integrator = md

tinit = 0.0

dt = 0.020

nsteps = 50000000

nstxout = 5000

nstvout = 5000

nstfout = 5000

nstlog = 5000

nstenergy = 5000

nstxout-compressed = 5000

compressed-x-precision = 100

cutoff-scheme = Verlet

ns_type = grid

nstlist = 20

pbc = xyz

verlet-buffer-tolerance = 0.005

epsilon_r = 15

coulombtype = reaction-field

rcoulomb = 1.1

vdw_type = cutoff

vdw-modifier = Potential-shift-verlet

rvdw = 1.1

tcoupl = v-rescale

tc-grps = protein solute

tau_t = 1.0 1.0

ref_t = 310.15 310.15

pcoupl = Parrinello-rahman

pcoupltype = isotropic

tau_p = 12.0

compressibility = 4.5e-5

ref_p = 1.0

gen_vel = no

continuation = yes

lincs_iter = 2

lincs_order = 4

Topology system.top

#include "toppar/martini_v3.0.0.itp"

#include "toppar/martini_v3.0.0_ions_v1.itp"

#include "toppar/martini_v3.0.0_nucleobases_v1.itp"

#include "toppar/martini_v3.0.0_phospholipids_v1.itp"

#include "toppar/martini_v3.0.0_phospholipids_v1_matthieu.itp"

#include "toppar/martini_v3.0.0_small_molecules_v1.itp"

#include "toppar/martini_v3.0.0_solvents_v1.itp"

#include "toppar/martini_v3.0.0_sugars_v1.itp"

#include "toppar/martini_v3.0_sterols_v1.0.itp"

#include "76finalmut_p6_proa.itp"

#include "76finalmut_p6_prob.itp"

[ system ]

; name

Martini system

[ molecules ]

; name number

PROA 1

PROB 1

W 151489

NA 1695

CL 1678
